# Development of tacrine clusters as positively cooperative systems for the inhibition of acetylcholinesterase

**DOI:** 10.1080/14756366.2021.1954917

**Published:** 2021-07-22

**Authors:** Tereza Cristina Santos Evangelista, Óscar López, Sabrina Baptista Ferreira, José G. Fernández-Bolaños, Magne O. Sydnes, Emil Lindbäck

**Affiliations:** a Department of Chemistry, Bioscience and Environmental Engineering, Faculty of Science and Technology, University of Stavanger, Stavanger, Norway; b Department of Organic Chemistry, Chemistry Institute, Federal University of Rio de Janeiro, Rio de Janeiro, Brazil; c Departamento de Química Orgánica, Facultad de Química, Universidad de Sevilla, Seville, Spain

**Keywords:** Acetylcholinesterase, CuAAC, multivalent interactions, multivalent inhibition potency, tacrine

## Abstract

The synthesis of four tetra-tacrine clusters where the tacrine binding units are attached to a central scaffold *via* linkers of variable lengths is described. The multivalent inhibition potencies for the tacrine clusters were investigated for the inhibition of acetylcholinesterase. Two of the tacrine clusters displayed a small but significant multivalent inhibition potency in which the binding affinity of each of the tacrine binding units increased up to 3.2 times when they are connected to the central scaffold.

## Introduction

Multivalent interactions (or multivalency) constitute a widespread recognition phenomenon in living organisms to establish interactions between carbohydrates and proteins, which are essential for the adhesion of viruses and bacteria to the surface of a cell in addition to cell adhesion^1^. The power of multivalent interactions is that when several binding modules are connected to a central scaffold and bind cooperatively to a target, the binding affinity of the multivalent ligand on a valency-corrected basis (rp/*n*) can be dramatically increased (i.e. the binding affinity of the multivalent ligands is stronger than the sum of its mono-valent ligands alone), which is known as the cluster effect or multivalent effect^2^. A well-researched field in bioorganic chemistry is the synthesis of multivalent glycoconjugates for investigation of the multivalent effect for carbohydrate-protein (lectin) interactions^2^^b^^,^^3^. On a valency-corrected basis, such multivalent assemblies of carbohydrates have achieved an affinity enhancement of an astonishing six orders of magnitude for the binding to lectins^4^. A much less explored field is multivalent enzyme inhibition, which has been associated with the fact that most enzymes possess a single deep active site that is expected to be less accessible for multimeric ligands than several binding pockets on the surface of lectins^5^. In fact, such pockets on the surface of lectins give rise to efficient chelating binding with multivalent glycoconjugates^6^, and therefore multivalent effect for enzyme inhibition has been disregarded^5^^b^. To the best of our knowledge, if we neglect bivalent enzyme inhibitors, multivalent enzyme inhibition potency has only been achieved for a few groups of enzymes including, glycosidases^2^^a,^^3^^a,^^7^, glycosyltransferases^8^, carbonic anhydrases^9^, and very recently for cholinesterases^10^. In this context, it is worth mentioning that a 36 valent inhibitor has been demonstrated to give rise to an astonishing affinity enhancement of ca 4700-fold on a valency-corrected basis for the inhibition of α-mannosidase^2^^a,^^11^, which emphasise the power of multivalent enzyme inhibition. However, there is no general linear correlation between valency and enzyme inhibition potency on a valency-corrected basis as observations have been made in which the inhibition on valency-corrected basis decrease by valency^9^^a,^^12^. Another parameter to consider in the design of efficient multivalent inhibitors is the choice of the scaffold where various types of scaffolds implement different spatial orientations of the inhitopes, which can affect the inhibition^8^^,^^13^. The length of the linkers connecting the central scaffold with its inhitopes has also been identified as an important parameter for efficient multivalent enzyme inhibition^14^.

**Figure 1.**
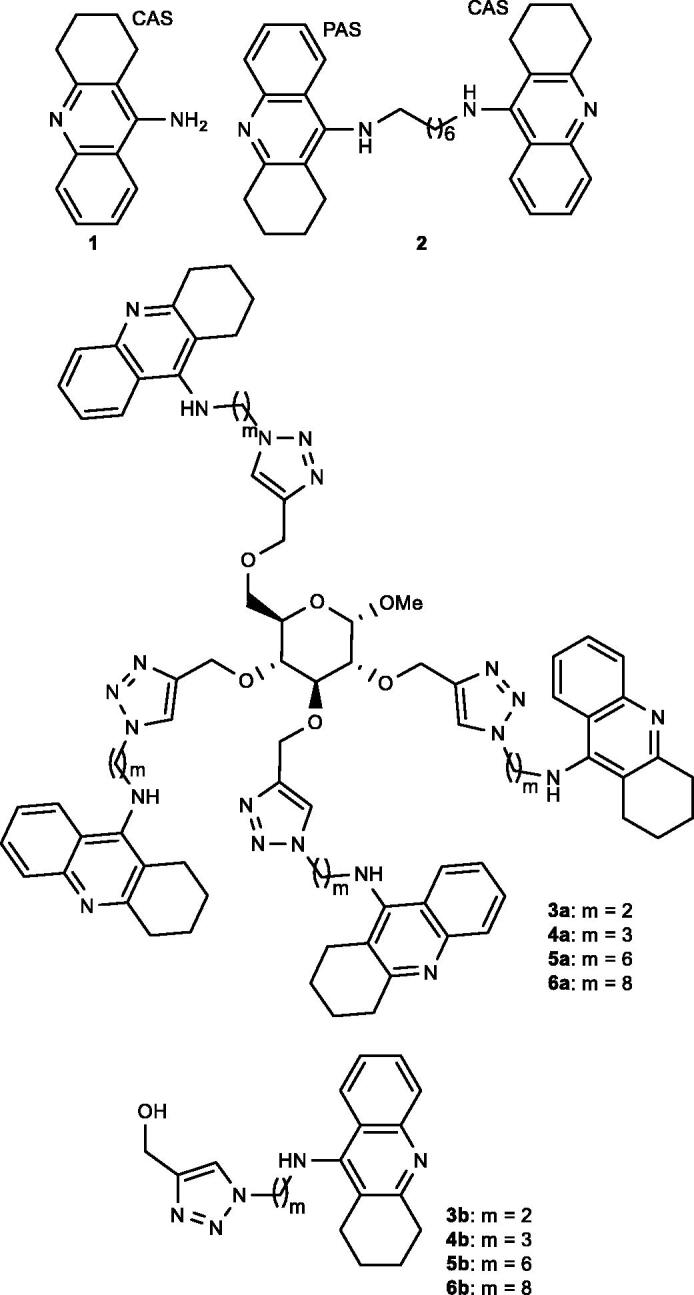
Illustration of known AChE inhibitors **1** and **2** and the tetravalent architectures **3a–6a**, which are the target molecules in this paper.

Alzheimer’s disease (AD) is a multifactorial progressive neurological disorder that represents the most common form of dementia^15^. Currently, there is no cure available for this devastating disease due to a lack of exact knowledge of its causes^16^. The cholinergic hypothesis suggests that the level of the neurotransmitter acetylcholine (ACh) is insufficient in the Alzheimer brain, which causes cognitive loss^17^. Therefore, inhibition of cholinesterases [acetylcholinesterase (AChE) and butyrylcholinesterase (BuChE)] and thereby increasing the concentration of ACh in the brain is an attractive target for the treatment of AD^18^. One example of a cholinesterase inhibitor drug for palliative treatment of AD is tacrine (**1**) (Figure 1), which unfortunately was discontinued in 2013 as it results in liver damage^19^. When the structure of AChE was solved by X-ray crystallography^20^, an active gorge, lined with aromatic residues, penetrating ca 20 Å into the enzyme was recognised hosting: (1) the active site including the catalytic triad and catalytic anionic binding subsite (CAS) nearby the bottom of the active gorge and (2) the peripheral anionic binding site (PAS) located at the interface of the active gorge.

When the structure of tacrine complexed with AChE was solved by X-ray crystallography, it was concluded that it binds to CAS in the solid state^21^. To establish interactions with both CAS and PAS, a bivalent strategy was pursued in which two tacrine rings were connected *via* a heptamethylene linker to obtain bis(7)tacrine (**2**) (Figure 1) that is a ca 1000-fold stronger AChE inhibitor than tacrine, which was associated with simultaneous interactions with CAS and PAS^22^. The finding of the enhanced AChE inhibition by bis(7)tacrine (**2**) triggered an avalanche of reported bivalent AChE inhibitors^18^^,^^23^.

**Figure 2.**
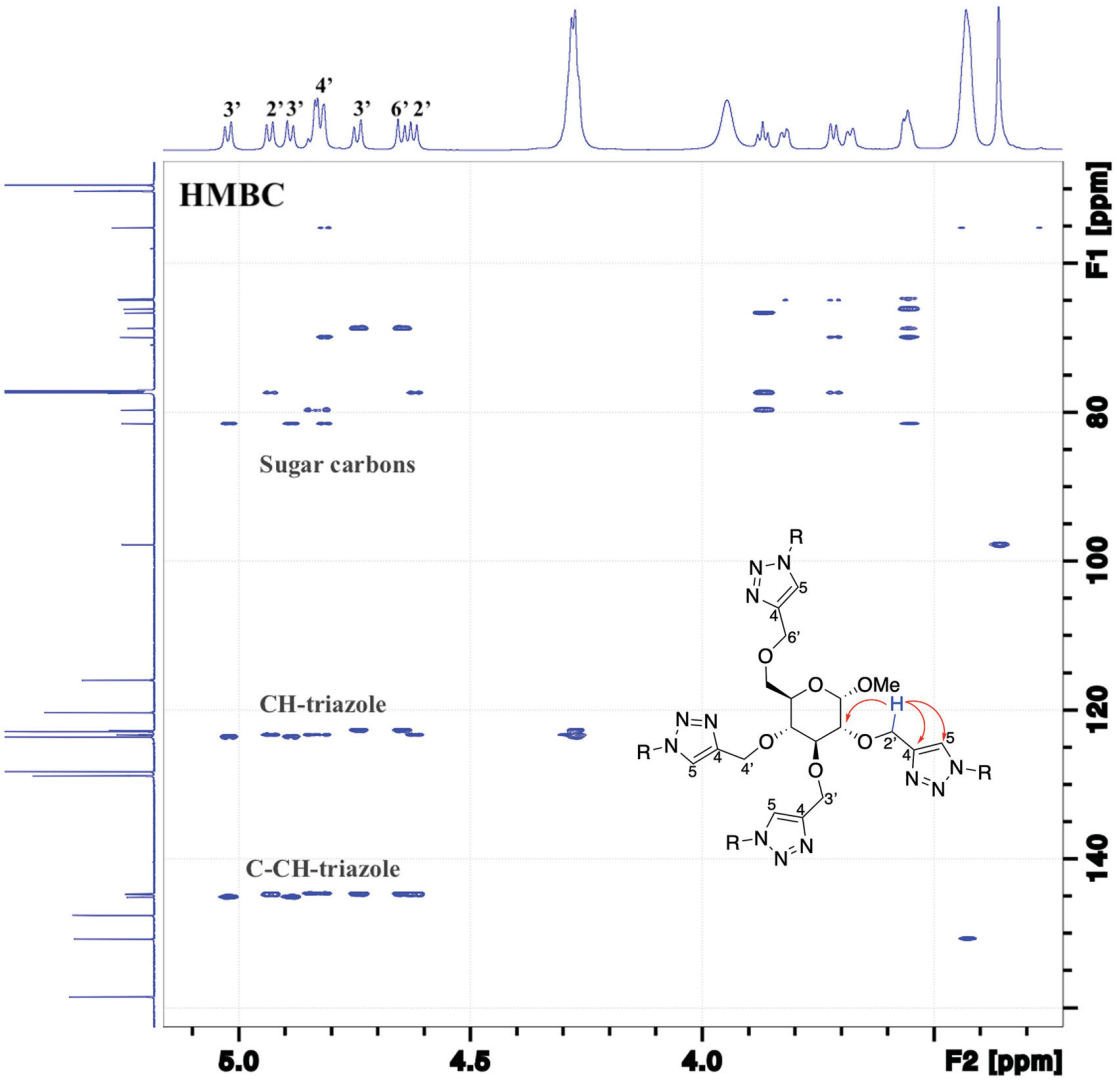
Part of the HMBC NMR spectra of tetravalent triazole tacrine **6a** in CDCl_3_ (850.13 MHz) (CH-triazole = C-5 carbons and C-CH-triazole = C-4 carbons).

The tetrameric structure of AChE that contains four catalytic subunits^24^ led us to propose tetra-tacrines **3a–6a** (Figure 1) as multivalent AChE inhibitors. We argued that when the tacrine rings are attached to the central sugar scaffold *via* linkers of optimal length they would employ the chelation effect^5^^b^ to bind simultaneously to the active gorges of the AChE tetramer to form a stable AChE: tetra-tacrine complex. An alternative mechanism to achieve multivalent inhibition by tetra-tacrines **3a–6a** is due to a statistical binding effect^5^^b^ caused by the increased effective concentration of the tacrine rings nearby the active gorges of AChE. Thus, in this paper we present: (1) the synthesis of the tetra-tacrines **3a–6a** (Figure 1), (2) the synthesis of the mono-tacrines **3b–6b**, and (3) the multivalent inhibition potencies of tetra-tacrines **3a–6a** against AChE by comparing them with reference compounds **3b–6b**.

## Materials and methods

### General procedures

DMF has dried over 4 Å molecular sieves (oven-dried). All reactions were carried out under an argon atmosphere unless otherwise specified. Microwave reactions were performed in a CEM Discover-SP, max power 300 W. TLC analyses were performed on Merck silica gel 60 F254 plates or Sigma-Aldrich aluminium oxide 60 F254 (neutral) plates using a UV light for detection. Silica gel NORMASIL 60^®^ 40–63 µm or Aluminium oxide Sigma–Aldrich 58 Å pore size was used for flash column chromatography. NMR spectra were recorded on a Bruker Avance NMR spectrometer; ^1^H NMR spectra were recorded at 400.13 or 850.13 MHz, ^13^C NMR spectra were recorded at 100.61 or 213.76 MHz, in CDCl_3_, MeOD, or DMSO. Chemical shifts are reported in ppm relative to an internal standard of residual chloroform (*δ* = 7.26 for ^1^H NMR; *δ* = 77.16 for ^13^C NMR), residual methanol (*δ* = 3.31 for ^1^H NMR; *δ* = 49.00 for ^13^C NMR) or residual DMSO (*δ* = 2.50 for ^1^H NMR; *δ* = 39.52 for ^13^C NMR). High-resolution mass spectra (HRMS) were recorded from on a Qexactive spectrometer in positive electrospray ionisation (ESI) mode.

### Synthetic protocols

#### General procedure for the preparation of compounds 3b–6b

A mixture of propargyl alcohol (**7)** (2.4 mmol, 7 equiv.), azide **8**, **9**, **10**, or **11** (0.2 mmol, 1 equiv.), and copper (II) sulphate pentahydrate (0.3 equiv.) in DMF (3 ml) in a foil-covered round bottom flask was added sodium ascorbate (0.6 equiv.). The mixture was kept stirring at room temperature overnight under Ar atmosphere. The solvent was then removed under reduced pressure and the concentrate was purified by silica gel flash column chromatography.

#### General procedure for the preparation of compounds 3a-6a

A mixture of the alkyne **13** (0.2 mmol, 69.3 mg, 1 equiv.), azide **8**, **9**, **10**, or **11** (4.8 mmol, 1.2 equiv. per reactive group of the alkyne), and copper (II) sulphate pentahydrate (0.3 equiv. per reactive group of the alkyne) in DMF (5 ml) was added sodium ascorbate (0.6 equiv. per reactive group of the alkyne). The mixture was irradiated in a microwave at 300 W and 115 °C for 45 min. Water (10 ml) was added and the crude mixture was extracted with dichloromethane (3 × 20 ml). The organic phases were combined, dried with MgSO_4_, and filtered. Evaporation of the solvent by reduced pressure yielded a crude material that was purified by column chromatography.

#### Cholinesterase assays

For the assessment of enzymatic inhibition, commercially available acetylcholinesterase from *Electrophorus electricus* (type V-S, Sigma Aldrich) was used, conducting minor modifications on Ellman’s protocol^25^. Stock solutions of inhibitors were prepared in DMSO, being the solvent content of 1.25% (V/V) in the final assay solutions. Enzymatic activities were measured in a UV–Vis instrument (Hitachi U-2900) using PS cuvettes containing 0.1 mM phosphate buffer (pH 8.0), 5,5′-dithiobis(2-nitrobenzoic acid) (DTNB, 0.88 mM, buffer solution), acetylthiocholine iodide as a model substrate, inhibitor, properly diluted aqueous enzyme solution, and water up to 1.2 ml. Solutions of the enzymes were prepared so as to keep the reaction rate within 0.12–0.15 Abs/min when [S] = 4 × *K_M_*. The formation of the chromophore was monitored during 125 s at 405 nm and 25 °C.

Calculation of IC_50_ values was accomplished by plotting %I *vs.* log[I] and adjusting to a second-order equation. Substrate concentration was kept at 121 µM, using 2–4 independent assays, each of them, being run in duplicate.

For the calculation of the kinetic parameters of the free enzyme, and in the presence of **3a**, five different substrate concentrations, ranging from ¼ *K_M_* to 4 × *K_M_* were used. Cornish-Bowden method^26^ provided the mode of inhibition of **3a**; for that purpose, two different plots were used: 1/v *vs.* [I] (Dixon plot) and [S]/v *vs.* [I]. Mixed inhibition was found for such compound, which means that it binds both, the free enzyme (*K_ia_*) and the enzyme-substrate complex (*K_ib_*). Kinetic parameters (*K_M_*, *V_max_*, *K_M_* app, *V_max app_*) were obtained through non-linear regression analysis (least squares fit) using the GraphPad Prism 8.01 software and inhibition constants were calculated using the following equations:

(1) $$K_{M},app=K_{M}\frac{1+\frac{\left[I\right]}{K_{ia}}}{1+\frac{\left[I\right]}{K_{ib}}}$$

(2) $$V_{\max\mathrm{app}}=\frac{V_{\max}}{1+\frac{[I]}{K_{ib}}}$$

Data are expressed as the mean ± *SD*.

## Results and discussion

### Synthesis

**Scheme 1.**
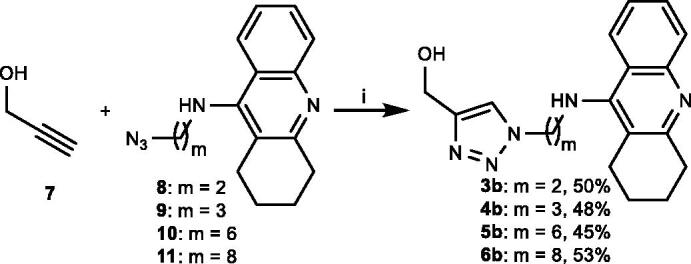
Synthesis of reference compounds **3b–6b**. (i) CuSO_4_·5H_2_O, sodium ascorbate, DMF, RT.

The presence of 1,2,3-triazole moieties in the linker between the pharmacophores in bivalent cholinesterase inhibitors has been found to establish interactions with residues in AChE^27^. Therefore, we considered it unsuitable to employ tacrine (**1**) as a reference compound for the evaluation of the multivalent inhibition potency of **3a–6a**. Instead, for each tetra-tacrine **3a**, **4a**, **5a**, and **6a** a mono-tacrine reference compound **3b**, **4b**, **5b**, and **6b**, respectively, was prepared to contain the 1,2,3-triazole moiety and the same number of CH_2_-groups between the tacrine ring and the hydroxyl group as the corresponding tetra-tacrine contains CH_2_-groups between its tacrine rings and central scaffold. Reference compounds **3b–6b** were obtained when propargyl alcohol (**7**) underwent Cu(I) catalysed alkyne-azide 1,3-dipolar cycloaddition (CuAAc) with azide armed tacrine derivatives **8**^28^ and **9–11**^23^^a^ (Scheme 1).

**Scheme 2.**
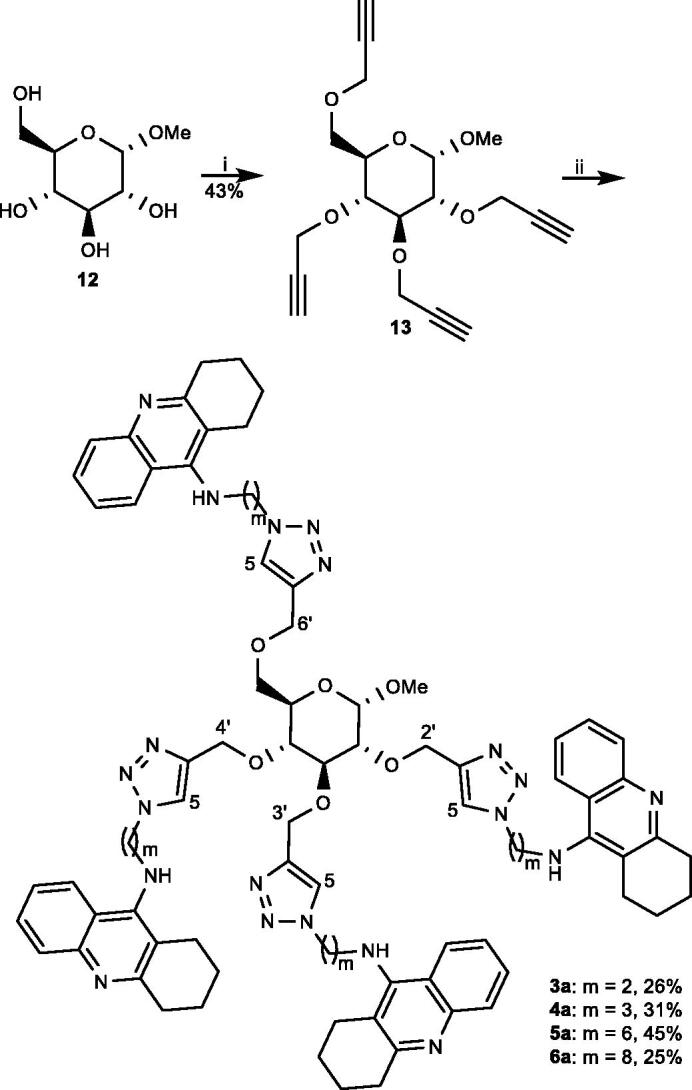
Synthesis of tetra-tacrines **3a–6a**. (i) NaH, propargyl bromide, DMF, RT, (ii) **8**, **9**, **10**, or **11**, CuSO_4_·5H_2_O, sodium ascorbate, DMF, MW, 115 °C.

The synthesis of the tetravalent tacrine architectures **3a–6a** commenced from commercially available methyl α-D-glucopyranoside (**12**), which was subjected to propargylation upon treatment with propargyl bromide and sodium hydride to provide **13** (Scheme 2). In the final step, tetra-alkyne **13** was armed with four tacrine inhitopes when it underwent Cu(I) catalysed alkyne-azide 1,3-dipolar cycloaddition with azides **8**, **9**, **10**, and **11** to obtain tetramers **3a**, **4a**, **5a**, and **6a**, respectively, with variable length of the linker between the central sugar scaffold and their inhitopes. The formation of 1,4-regiosisomeric triazole moieties in **3a–6a** was supported with ^13 ^C-NMR spectroscopy where the carbon atoms in 5-position in the triazole moieties consistently appeared in the range 124.8 to 122.7 ppm, which agrees with reported data for such isomers^29^. The carbons in 5-position (CH-triazole) were in turn identified through HMBC correlation with the CH_2_-protons (2′-H, 3′-H, 4′-H, and 6′-H) between the triazole moieties and the central sugar scaffold (Figure 2).

### Inhibition studies

The potency of tetra-tacrines **3a–6a** and mono-tacrines **3b–6b** for the inhibition of *Electrophorus electricus* AChE were tested using the Ellman method^25^ and the activities are presented in Table 1. All the tacrine-monomers **3b–6b** displayed potency in the nM concentration range from IC_50_= 566 nM down to IC_50_ = 7.1 nM for the inhibition of AChE. The mono-tacrines with longer linkers [**5b** (*m* = 6) and **6b** (*m* = 8)] between the tacrine and triazole rings are significantly stronger inhibitors than **3b** (*m* = 2) and **4b** (*m* = 3) with shorter linkers, which indicates that the longer ligands establish more efficient simultaneous interactions with PAS and CAS in the active gorge. The tetra-tacrines **3a–6a** also displayed potency in nM concentration range (IC_50_ = 12.5 nM to IC_50_ = 232 nM). However, for these tetra-valent inhibitors, there was no clear trend between the linker length between the tacrine and triazole rings as the strongest tetra-tacrine AChE inhibitor **5a** (IC_50_ = 12.5 nM, *m* = 6) behaves as a 36-fold stronger inhibitor than the weakest tetra-tacrine inhibitor **6a** (IC_50_ = 232 nM, *m* = 8).

**Table 1.** Relative inhibition potencies (rp), inhibition potencies on valency-corrected basis (rp/*n*) for tetra-tacrines **3a–6a** and inhibitory potencies (IC_50_ [nM]) against *Electrophorus electricus* AChE by **3a–6a** and **3b–6b**.

Inhibitor	AChE IC_50_^a^	AChE rp^b^	AChE rp/*n*^c^
**3a**	43.7 ± 7.3 nM	12.9	3.2
**3b**	565 ± 79 nM	—	—
**4a**	60.2 ± 5.5 nM	5.8	1.5
**4b**	348 ± 23 nM	—	—
**5a**	12.5 ± 3.3 nM	1.0	0.25
**5b**	12.6 ± 2.4 nM	—	—
**6a**	232 ± 21 nM	0.03	0.008
**6b**	7.1 ± 1.0 nM	—	—
Tacrine	53.4 ± 1.1 nM	—	—
Methyl α-D-glucopyranoside	N.I.^d^	—	—

^a^[S] = 121 μM (S = substrate).

^b^rp = IC_50_ (mono-tacrine)/IC_50_ (tetra-tacrine).

^c^rp/*n* = rp/number of tacrine rings.

^d^Tested at 100 μM inhibitor concentration.

**Figure 3.**
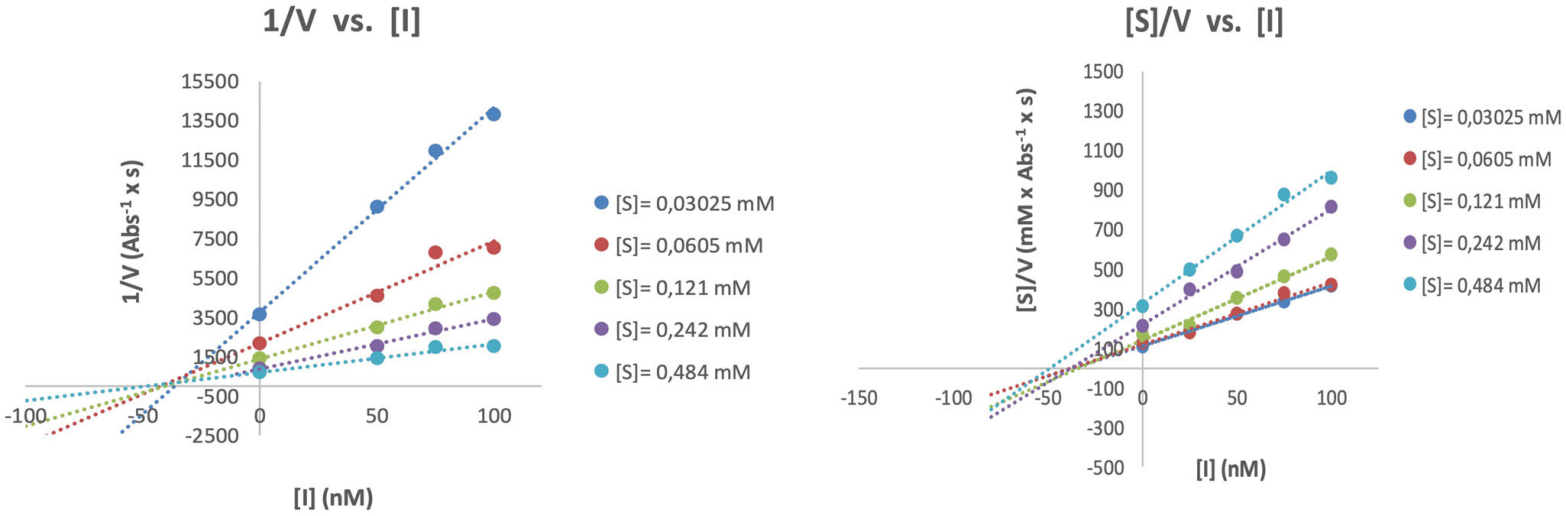
Cornish-Bowden plots for compound **3a** against electrophorus electricus AChE (V: rate of reaction; [S]: substrate concentration; [I]: inhibitor concentration).

The relative inhibition potency (rp) was obtained by dividing the IC_50_ value of the mono-tacrine with the IC_50_ value of the corresponding tetra-tacrine, which contains the same number of CH_2_-groups between the tacrine and triazole rings [for instance, rp = IC_50_(**3b**)/IC_50_(**3a**) = 12.9]. The relative inhibition potencies for tetra-tacrines **3a–6a** demonstrates that longer linkers between the triazole and tacrine rings have a destructive impact on the inhibition potency, as the rp-values gradually decrease from rp = 12.9 for **3a** (*m* = 2) to rp = 0.03 for **6a** (*m* = 8). The inhibition potencies on valency-corrected basis (rp/*n*) showed that tetra-tacrines **3a** (rp/*n* = 3.2, *m* = 2) and **4a** (rp/*n* = 1.5, *m* = 3) exhibit small but significant multivalent inhibition potencies for AChE. The rp/*n*-values for **5a** (rp/*n* = 0.25, *m* = 6) and **6a** (rp/*n* = 0.008, *m* = 8) on the other hand demonstrate that the mono-tacrines **5b** (*m* = 6) and **6b** (*m* = 8) were 75% and more than 99% less active, respectively, when they are connected to the central multivalent sugar scaffold. From a Cornish-Bowden plot (Figure 3) for **3a**, we concluded that it causes a mixed inhibition mode of AChE [*K_ia_* = 31.6 ± 2.0 nM (competitive inhibition constant) and *K_ib_* = 45.0 ± 5.9 nM (non-competitive inhibition constant)], which implies that it binds to the catalytic site in addition to a second binding site, for instance, PAS on the entrance of the active gorge. Thus, the multivalent inhibitory potency observed for **3a** and **3b** might be due to the chelation effect in which the length of the linkers in **3a** and **4a** are of sufficient length to allow simultaneous binding of their tacrine inhitopes to more than one active gorge in the tetrameric AChE enzyme. On the other hand, shorter linkers in the tetra-tacrines imply higher effective concentration nearby the active gorges, and thus a statistical binding effect cannot be excluded as the reason for the observed multivalent inhibition potency observed for tetra-tacrines **3a** and **4a**. However, as rp/*n* ˂ 1 for **5a** and **6a**, in such statistical binding effect scenario, it implies that another effect is involved, which oppose the binding of the inhitopes to the enzyme for example that longer linkers affect the position of the tacrine rings in such a way that they become less accessible for the enzyme.

## Conclusions

We have applied the Cu(I)-catalysed azide–alkyne Huisgen cycloaddition reaction to obtain four tetra-tacrine clusters **3a–6a** in which the tacrine rings are connected to a central scaffold *via* linkers of variable lengths. Two of the tetra-tacrines **3a** and **4a** with the shortest linkers displayed a small but significant multivalent effect in the inhibition of AChE. The observed multivalent inhibition potency is proposed to arise from the chelation or statistical binding effects.

## Supplementary Material

Supplemental Material

## References

[1] Mammen M, Choi SK, Whitesides GM. Polyvalent interactions in biological systems: implications for design and use of multivalent ligands and inhibitors. Angew Chem Int Ed 1998;37:2754–94. 10.1002/(SICI)1521-3773(19981102)37:20<2754::AID-ANIE2754>3.0.CO;2-3 29711117

[2] (a) Compain P. Multivalent effect in glycosidase inhibition: the end of the beginning. Chem Rec 2020;20:10–22. 30993894 10.1002/tcr.201900004

[3] (a) González-Cuesta M, Mellet CO, Fernández JMG. Carbohydrate supramolecular chemistry: beyond the multivalent effect. Chem Commun 2020;56:5207–22. 10.1039/d0cc01135e 32322844

[4] (a) Marra A, Staderini S, Berthet N, et al. Thiyl glycosylation of propargylated octasilsesquioxane: synthesis and lectin-binding properties of densely glycosylated clusters on a cubic platform. Eur J Org Chem 2013;2013:1144–9.

[5] (a) Nierengarten I, Nierengarten JF. Fullerene sugar balls: a new class of biologically active fullerene derivatives. Chem Asian J 2014;9:1436–44. 24678063 10.1002/asia.201400133

[6] Fasting C, Schalley CA, Weber M, et al. Multivalency as a chemical organization and action principle. Angew Chem Int Ed Engl 2012;51:10472–98. 22952048 10.1002/anie.201201114

[7] (a) Assailly C, Bridot C, Saumonneau A, et al. Polyvalent transition-state analogues of sialyl substrates strongly inhibit bacterial sialidases. Chemistry 2021;27:3142–50. 33150981 10.1002/chem.202004672

[8] Tikad A, Fu H, Sevrain CM, et al. Mechanistic insight into heptosyltransferase inhibition by using Kdo multivalent glycoclusters. Chemistry 2016;22:13147–55. 27516128 10.1002/chem.201602190

[9] (a) Carta F, Osman SM, Vullo D, et al. Dendrimers incorporating benzenesulfonamide moieties strongly inhibit carbonic anhydrase isoforms I-XIV. Org Biomol Chem 2015;13:6453–7. 25976058 10.1039/c5ob00715a

[10] (a) Zhao S, Xu J, Zhang S, et al. Multivalent butyrylcholinesterase inhibitor discovered by exploiting dynamic combinatorial chemistry. Bioorg Chem 2021;108:104656. 33548731 10.1016/j.bioorg.2021.104656

[11] Lepage ML, Schneider JP, Bodlenner A, et al. Iminosugar-cyclopeptoid conjugates raise multivalent effect in glycosidase inhibition at unprecedented high levels. Chemistry 2016;22:5151–5. 26917097 10.1002/chem.201600338

[12] (a) Joosten A, Schneider JP, Lepage ML, et al. A convergent strategy for the synthesis of second‐generation iminosugar clusters using “clickable” trivalent dendrons. Eur J Org Chem 2014;2014:1866–72.

[13] Brissonnet Y, Mellet CO, Morandat S, et al. Topological effects and binding modes operating with multivalent iminosugar-based glycoclusters and mannosidases. J Am Chem Soc 2013;135:18427–35. 24224682 10.1021/ja406931w

[14] (a) Schneider JP, Tommasone S, Sala PD, et al. Synthesis and glycosidase inhibition properties of calix[8]arene-based iminosugar click clusters. Pharmaceuticals 2020;13:366. 10.3390/ph13110366 PMC7694328 33167387

[15] Alzheimer's Association. 2016 Alzheimer's disease facts and figures. Alzheimers Dement 2016;12:459–509. 27570871 10.1016/j.jalz.2016.03.001

[16] Craig LA, Hong NS, McDonald RJ. Revisiting the cholinergic hypothesis in the development of Alzheimer's disease. Neurosci Biobehav Rev 2011;35:1397–409. 21392524 10.1016/j.neubiorev.2011.03.001

[17] Bartus RT, Dean RL, III, Beer B, Lippa AS. The cholinergic hypothesis of geriatric memory dysfunction. Science 1982;217:408–14. 7046051 10.1126/science.7046051

[18] Anand P, Singh B. A review on cholinesterase inhibitors for Alzheimer's disease. Arch Pharm Res 2013;36:375–99. 23435942 10.1007/s12272-013-0036-3

[19] Sharma K. Cholinesterase inhibitors as Alzheimer's therapeutics. Mol Med Rep 2019;20:1479–87. 31257471 10.3892/mmr.2019.10374 PMC6625431

[20] Sussman JL, Harel M, Frolow F, et al. Atomic structure of acetylcholinesterase from Torpedo californica: a prototypic acetylcholine-binding protein. Science 1991;253:872–9. 1678899 10.1126/science.1678899

[21] Harel M, Schalk I, Ehret-Sabatier L, et al. Quaternary ligand binding to aromatic residues in the active-site gorge of acetylcholinesterase. Proc Natl Acad Sci USA 1993;90:9031–5. 8415649 10.1073/pnas.90.19.9031 PMC47495

[22] Pang YP, Quiram P, Jelacic T, et al. Highly potent, selective, and low cost bis-tetrahydroaminacrine inhibitors of acetylcholinesterase. Steps toward novel drugs for treating Alzheimer's disease. J Biol Chem 1996;271:23646–9. 8798583 10.1074/jbc.271.39.23646

[23] (a) de Santana QLO, Evangelista TCS, Imhof P, et al. Tacrine-sugar mimetic conjugates as enhanced cholinesterase inhibitors. Org Biomol Chem 2021;19:2322–37. 33645607 10.1039/d0ob02588g

[24] (a) Bourne Y, Grassi J, Bougis PE, Marchot P. Conformational flexibility of the acetylcholinesterase tetramer suggested by X-ray crystallography. J Biol Chem 1999;274:30370–6. 10521413 10.1074/jbc.274.43.30370

[25] Ellman GL, Courtney KD, Andres V, Feather-Stone RM. A new and rapid colorimetric determination of acetylcholinesterase activity. Biochem Pharmacol 1961;7:88–95. 13726518 10.1016/0006-2952(61)90145-9

[26] Cornish-Bowden A. A simple graphical method for determining the inhibition constants of mixed, uncompetitive and non-competitive inhibitors. Biochem J 1974;137:143–4. 4206907 10.1042/bj1370143 PMC1166095

[27] Najafi Z, Mahdavi M, Saeedi M, et al. Novel tacrine-coumarin hybrids linked to 1,2,3-triazole as anti-Alzheimer's compounds: *in vitro* and *in vivo* biological evaluation and docking study. Bioorg Chem 2019;83:303–16. 30396115 10.1016/j.bioorg.2018.10.056

[28] Oukoloff K, Coquelle N, Bartolini M, et al. Design, biological evaluation and X-ray crystallography of nanomolar multifunctional ligands targeting simultaneously acetylcholinesterase and glycogen synthase kinase-3. Eur J Med Chem 2019;168:58–77. 30798053 10.1016/j.ejmech.2018.12.063

[29] Creary X, Anderson A, Brophy C, et al. Method for assigning structure of 1,2,3-triazoles. J Org Chem 2012;77:8756–61. 22894557 10.1021/jo301265t

