# Supplementary material for: Development of tacrine clusters as positively cooperative systems for the inhibition of acetylcholinesterase
Source: J Enzyme Inhib Med Chem. 2021 Jul 22;36(1):1659–64. doi: 10.1080/14756366.2021.1954917 (PMC8317962; doi:10.1080/14756366.2021.1954917)

Development of Tacrine Clusters as Positively Cooperative Systems for the Inhibition of Acetylcholinesterase

Tereza Cristina Santos Evangelista[a,b], Óscar López[c], Sabrina Baptista Ferreira[b], José G. Fernández-Bolaños[c], Magne O. Sydnes[a], and Emil Lindbäck[a*]

[a] *Department of Chemistry, Bioscience and Environmental Engineering, Faculty of Science and Technology, University of Stavanger, NO-4036 Stavanger, Norway E-mail: emil.lindback@uis.no*

[b] *Department of Organic Chemistry, Chemistry Institute, Federal University of Rio de Janeiro, 21949-900 Rio de Janeiro, Brazil*

[c] *Departamento de Química Orgánica, Facultad de Química, Universidad de Sevilla c/Profesor García González 1, 41012 Seville, Spain*

Table of contents

1. **Synthesis of Methyl 2,3,4,6-Tetra-O-propargyl-α-D-glucopyranoside (13)** **3**

2. **Compounds 3b-6b** **3**

   2.1. **(1-(2-((1,2,3,4-tetrahydroacridin-9-yl)amino)ethyl)-1*H*-1,2,3-triazol-4-yl)metanol (3b)** **3**

   2.2. **(1-(3-((1,2,3,4-tetrahydroacridin-9-yl)amino)propyl)-1*H*-1,2,3-triazol-4-yl)metanol (4b)** .. **4**

   2.3. **(1-(6-((1,2,3,4-tetrahydroacridin-9-yl)amino)hexyl)-1*H*-1,2,3-triazol-4-yl)metanol (5b)** **4**

   2.4. **(1-(8-((1,2,3,4-Tetrahydroacridin-9-yl)amino)octyl)-1*H*-1,2,3-triazol-4-yl)metanol (6b)** **5**

3. **Compounds 3a-6a** **5**

   3.1. **Compound 3a** **5**

   3.2. **Compound 4a** **6**

   3.3. **Compound 5a** **6**

   3.4. **Compound 6a** **7**

4. **References** **8**

5. **NMR spectra** **9**

1. Synthesis of methyl 2,3,4,6-tetra-*O*-propargyl-α-D-glucopyranoside (13)

NaH 60% dispersion in mineral oil (910 mg, 22.8 mmol, 10 equiv.) was added in small portions to a solution of methyl α-D-glucopyranoside (410 mg, 2.28 mmol, 1 equiv.) in anhydrous DMF (20 mL) under an Ar atmosphere at 0 °C. The suspension was stirred for one hour followed by dropwise addition of propargyl bromide (80 wt% in toluene, 1.52 mL, 14 mmol, 6.1 equiv.). The reaction mixture was kept stirring at room temperature for 3 hours. After this time, the reaction was put on an ice-bath and 10 mL of MeOH was added. The solvent was removed under reduced pressure after 30 min. Water (10 mL) was added and the compound was extracted with diethyl ether (2 x 20 mL). The organic extracts were combined, dried ($MgSO_4$), filtered, and concentrated under reduced pressure. The crude material was purified by silica gel flash column chromatography (PE/EtOAc 90:10 → 80:20) to afford **13** (336 mg, 43%) as a yellow syrup. R_f 0.28 (PE/EtOAc 7:3); $[\alpha]_D^{25}+69$ (*c* 0.58, $CHCl_3$) {Lit.[1] $[\alpha]_D^{25}+80$ (*c* 1, $CHCl_3$)} ^{1}H NMR δ_H ($CDCl_3$, 400.13 MHz) 4.86 (d, 1H, $J_{5,4}$ = 3.5 Hz, 1-H), 4.49-4.37 (m, 4H, CH_2CCH), 4.30-4.14 (m, 4H, CH_2CCH), 3.82-3.77 (m, 2H, 3-H and 6a-H), 3.73-3.65 (m, 2H, 6b-H and 5-H), 3.58 (dd, 1H, $J_{6a,6b}$ = 9.6 Hz and $J_{6a,1}$ = 3.5 Hz, 2-H), 3.47 (t, 1H, $J_{6b,6a}$ = 9.4 Hz, 4-H), 3.37 (s, 3H, OCH_3), 2.43-2.40 (m, 4H, CCH); ^{13}C NMR δ_C ($CDCl_3$, 100.61 MHz) 98.1 (1-C), 81.5 (3-C), 80.2 (CCH), 79.9 (CCH), 79.5 (CCH), 79.3 (2-C), 76.7 (4-C), 75.1 (CH), 75.1 (CH), 74.5 (CH), 74.4 (CH), 69.6 (5-C), 68.2 (6-C), 60.5 (CH_2), 60.3 (CH_2), 58.8 (CH_2), 58.7 (CH_2), 55.4 (OCH_3).

The NMR data for **13** was in agreement with that reported data.[1]

2. Compounds 3b-6b

2.1. (1-(2-((1,2,3,4-tetrahydroacridin-9-yl)amino)ethyl)-1*H*-1,2,3-triazol-4-yl)metanol (3b)

The crude product of **3b** was purified by aluminum oxide flash column chromatography ($CH_3CN/H_2O/NH_4OH$ 99:1:0.1 → 98:2:0.1) to provide **3b** (55.5 mg, 50%) as a yellow solid. R_f 0.61 (Aluminum oxide: $CH_3CN/H_2O/NH_4OH$ 9:1:0.1); ^{1}H NMR δ_H (DMSO, 400.13 MHz) 7.98 (d, 1H, $J_{8,7}$ = 8.4 Hz, 8-H), 7.88 (s, 1H, ArH-triazole), 7.74 (d, 1H, $J_{5,6}$ = 8.4 Hz, 5-H), 7.58 (t, 1H, $J_{6,7}$ = 7.4 Hz, 6-H), 7.37 (t, 1H, $J_{7,6}$ = 7.6 Hz, 7-H), 5.12 (t, 1H, J = 5.6 Hz, OH), 4.59 (t, 2H, J = 6.2 Hz, CH_2), 4.46 (d, 2H, J = 5.5 Hz, CH_2-OH), 3.93-3.88 (m, 2H, CH_2-NH), 2.91 (t, 2H, $J_{4,3}$ = 6.3 Hz, 4-H), 2.61 (t, 2H, $J_{1,2}$ = 6.3 Hz, 1-H), 1.83-1.76 (m, 4H, 2-H and 3-H); ^{13}C NMR δ_C (DMSO, 100.61 MHz) 156.7 (Ar), 150.8 (Ar), 147.8 (Ar), 144.9 (Ar), 128.9 (6-C), 126.4 (5-C),

123.9 (8-C), 123.2 (7-C), 123.2 (CH-triazole), 119.4 (Ar), 116.0 (Ar), 55.0 (CH_2-OH), 49.5 (CH_2), 47.7 (CH_2-NH), 32.3 (4-C), 24.6 (1-C), 22.3 (2-C or 3-C), 21.9 (2-C or 3-C); HRMS (ESI): Calcd. for $C_{18}H_{22}N_5O^+$ $[M + H]^+$ 324.1819; found 324.1816.

2.2. (1-(3-((1,2,3,4-tetrahydroacridin-9-yl)amino)propyl)-1*H*-1,2,3-triazol-4-yl)metanol (4b)

The crude product of **4b** was purified by silica gel flash column chromatography ($CH_3CN/H_2O/NH_4OH$ 95:5:0.1 → 94:6:0.1) to provide **4b** (50.0 mg, 48%) as a yellow foam. R_f 0.23 ($CH_3CN/H_2O/NH_4OH$ 99:1:0.1); ^1H NMR δ_H (MeOD, 850.13 MHz) 8.02 (d, 1H, $J_{8,7}$ = 8.4 Hz, 8-H), 7.85 (s, 1H, ArH-triazole), 7.76 (d, 1H, $J_{5,6}$ = 8.3 Hz, 5-H), 7.58-7.56 (m, 1H, 6-H), 7.38-7.36 (m, 1H, 7-H), 4.65 (s, 2H, CH_2-OH), 4.49 (t, 2H, J = 6.7 Hz, CH_2), 3.54 (t, 2H, J = 7.0 Hz, CH_2-NH), 2.97 (t, 2H, $J_{4,3}$ = 6.3 Hz, 4-H), 2.69 (t, 2H, $J_{1,2}$ = 6.2 Hz, 1-H), 2.25 (quint, 2H, J = 6.9 Hz, CH_2), 1.91-1.86 (m, 4H, 2-H and 3-H); ^{13}C NMR δ_C (MeOD, 213.76 MHz) 158.5 (Ar), 153.4 (Ar), 149.2 (Ar), 146.8 (Ar), 130.3 (6-C), 127.1 (5-C), 125.3 (8-C), 124.3 (7-C), 124.2 (CH-triazole), 121.0 (Ar), 117.2 (Ar), 56.4 (CH_2-OH), 48.8 (CH_2), 46.3 (CH_2-NH), 33.6 (4-C), 32.4 (CH_2), 26.1 (1-C), 23.9 (2-C or 3-C), 23.4 (2-C or 3-C); HRMS (ESI): Calcd. for $C_{19}H_{24}N_5O^+$ $[M + H]^+$ 338.1975; found 338.1974.

2.3. (1-(6-((1,2,3,4-tetrahydroacridin-9-yl)amino)hexyl)-1*H*-1,2,3-triazol-4-yl)metanol (5b)

The crude product of **5b** was purified by silica gel flash column chromatography ($CH_3CN/H_2O/NH_4OH$ 95:5:0.1 → 94:6:0.1 → 90:10:0.1) to provide **5b** (50.1 mg, 45%) as a brown syrup. R_f 0.38 ($CH_3CN/H_2O/NH_4OH$ 95:5:0.1); ^1H NMR δ_H (MeOD, 400.13 MHz) 8.10 (d, $J_{8,7}$ = 8.4 Hz, 1H, 8-H), 7.85 (s, 1H, ArH-triazole), 7.79 (d, 1H, $J_{5,6}$ = 8.4 Hz, 5-H), 7.58 (t, 1H, $J_{6,7}$ = 7.4 Hz, 6-H), 7.37 (t, 1H, $J_{7,6}$ = 7.6 Hz, 7-H), 4.66 (s, 2H, CH_2-OH), 4.32 (t, 2H, J = 7.0 Hz, CH_2), 3.56 (t, 2H, J = 7.2 Hz, CH_2-NH), 2.97 (t, 2H, $J_{4,3}$ = 5.2 Hz, 4-H), 2.68 (t, 2H, $J_{1,2}$ = 5.0 Hz, 1-H), 1.87-1.80 (m, 6H, 2-H, 3-H and CH_2), 1.67-1.59 (m, 2H, CH_2), 1.40-1.33 (m, 2H, CH_2), 1.30-1.23 (m, 2H, CH_2); ^{13}C NMR δ_C (MeOD, 100.61 MHz) 157.4 (Ar), 154.2 (Ar), 149.0 (Ar), 145.9 (Ar), 130.7 (6-C), 126.2 (5-C), 125.1 (8-C), 124.8 (7-C), 124.0 (CH-triazole), 120.2 (Ar), 115.8 (Ar), 56.5 (CH_2-OH), 51.1 (CH_2), 49.3 (CH_2-NH), 33.0 (4-C), 31.8 (CH_2), 31.1 (CH_2), 27.2 (CH_2), 27.1

(CH_2), 25.8 (1-C), 23.8 (2-C or 3-C), 23.2 (2-C or 3-C); HRMS (ESI): Calcd. for $C_{22}H_{30}N_5O^+$ [M + H]$^+$ 380.2425; found 380.2442.

2.4. (1-(8-((1,2,3,4-Tetrahydroacridin-9-yl)amino)octyl)-1*H*-1,2,3-triazol-4-yl)metanol (6b)

The crude product of **6b** was purified by silica gel flash column chromatography ($CH_3CN/H_2O/NH_4OH$ 95:5:0.1 → 94:6:0.1) to provide the title compound **6b** (57.0mg, 53%) as a yellow syrup. R_f 0.23 ($CH_3CN/H_2O/NH_4OH$ 90:10:0.1); ^1H NMR δ_H (MeOD, 400.13 MHz) 8.13 (d, 1H, $J_{8,7}$ = 8.6 Hz, 8-H), 7.86 (s, 1H, ArH-triazole), 7.76 (d, 1H, $J_{5,6}$ = 8.4 Hz, 5-H), 7.59 (t, 1H, $J_{6,7}$ = 7.6 Hz, 6-H), 7.39 (t, 1H, $J_{7,6}$ = 7.6 Hz, 7-H), 4.66 (s, 2H, CH_2-OH), 4.35 (t, 2H, J = 7.0 Hz, CH_2), 3.58 (t, 2H, J = 7.1 Hz, CH_2-NH), 2.98 (t, 2H, $J_{4,3}$ = 5.5 Hz, 4-H), 2.74 (t, 2H, $J_{1,2}$ = 5.8 Hz, 1-H), 1.94-1.89 (m, 4H, 2-H and 3-H), 1.88-1.81 (m, 2H, CH_2), 1.68-1.61 (m, 2H, CH_2), 1.36-1.22 (m, 8H, 4xCH_2); ^{13}C NMR δ_C (MeOD, 100.61 MHz) 158.1 (Ar), 154.0 (Ar), 149.0 (Ar), 146.8 (Ar), 130.4 (6-C), 126.8 (5-C), 124.9 (8-C), 124.7 (7-C), 124.0 (CH-triazole), 120.7 (Ar), 116.3 (Ar), 56.5 (CH_2-OH), 51.2 (CH_2), 49.6 (CH_2-NH overlaps with solvent signal), 33.5 (4-C), 32.1 (CH_2), 31.2 (CH_2), 30.1 (CH_2), 29.8 (CH_2), 27.2 (CH_2), 27.3 (CH_2), 26.0 (1-C), 23.9 (2-C or 3-C), 23.4 (2-C or 3-C); HRMS (ESI): Calcd. for $C_{14}H_{34}N_5O^+$ [M + H]$^+$ 408.2758; found 408.2758.

3. Compounds 3a-6a

3.1. Compound 3a

The crude product of **3a** was purified by aluminum oxide flash column chromatography (CH_2Cl_2/MeOH 99:1→ 98:2) to provide **3a** (65.8 mg, 26%) as a yellow foam. R_f 0.21 (Aluminum oxide: CH_2Cl_2/MeOH 99:1); $[\alpha]_D^{26}$ + 12 (c 0.17, $CHCl_3$); ^1H NMR δ_H ($CDCl_3$, 850.13 MHz) 8.00 (s, 1H, ArH-triazole), 7.89-7.88 (m, 4H, 4xArH), 7.86-7.84 (m, 2H, 2xArH-triazole), 7.77-7.73 (m, 4H, 4xArH), 7.68 (s, 1H, ArH-triazole), 7.51-7.48 (m, 3H, 3xArH), 7.46 (t, 1H, J = 7.5 Hz, ArH), 7.31-7.27 (m, 3H, 3xArH), 7.23 (t, 1H, J = 7.6 Hz, ArH), 4.92 (d, 1H, J = 11.2 Hz, CH_a-3'), 4.82-4.77 (m, 5H, 1-H, CH_b-3', CH_a-4', 2xNH), 4.74-4.70 (m, 4H, CH_b-4', CH_a-6', CH_a-2', NH), 4.59 (d, 1H, J = 12.6 Hz, CH_b-6'), 4.55-4.50 (m, 9H, CH_b-2', 4xCH_2), 3.96-3.92 (m, 8H, 4xCH_2-tacrine), 3.80-3.77 (m, 2H, 3-H, CH_a-6), 3.65 (d, 1H, J = 10.7 Hz, CH_b-6), 3.62 (d, 1H, J = 9.7 Hz, 5-H), 3.48-3.47 (m, 1H, 4-H), 3.45 (d, 1H, J = 9.5 Hz, 2-H), 3.36 (s, 3H, OCH_3), 3.02 (bs, 8H, 4xCH_2-tacrine), 2.60-2.54 (m, 8H, 4xCH_2-tacrine), 1.85-1.74 (m, 16H, 4xCH_2-tacrine, 4xCH_2); ^{13}C NMR δ_H ($CDCl_3$, 213.76 MHz) 158.5-158.4 (4xAr), 150.0-149.7 (4xAr), 146.7-146.6 (4xAr),

145.4 (Ar-triazole), 145.0-144.9 (3xAr-triazole), 128.9 (3xAr), 128.8 (2xAr), 128.3 (Ar), 128.2 (Ar), 128.0 (Ar), 124.8 (CH-triazole), 124.6 (CH-triazole), 124.5 (4xAr), 124.4 (CH-triazole), 124.1 (CH-triazole), 122.4-122.3 (4xAr), 120.5 (Ar), 120.4 (2xAr), 120.3 (Ar), 117.9-117.6 (4xAr), 97.6 (1-C), 81.4 (3-C), 79.6 (4-C), 77.4 (2-C), 70.0 (5-C), 68.7 (6-C), 66.4 (3'-C), 65.7 (2'-C), 64.7 (6'-C), 64.3 (4'-C), 55.4 (OCH_3), 50.8-50.7 (4xCH_2), 48.0-47.9 (4xCH_2-tacrine), 33.7-33.5 (4xCH_2-tacrine), 25.1 (3xCH_2-tacrine), 25.0 (CH_2-tacrine), 23.0-22.9 (4xCH_2-tacrine), 22.7-22.6 (4xCH_2); HRMS (ESI): Calcd. for $C_{79}H_{91}N_{20}O_6^+$ $[M + H]^+$ 1415.7425; found 1415.7400.

3.2. Compound 4a

The crude product of **4a** was purified by aluminum oxide flash column chromatography (CH_2Cl_2/MeOH 99:1 → 98:2) to provide **4a** (73.3 mg, 31%) as a brown foam. R_f 0.25 (Aluminum oxide: CH_2Cl_2/MeOH/NH_4OH 99:1:0.1); $[\alpha]_D^{26}$ + 33 (*c* 0.12, $CHCl_3$); ^1H NMR δ_H ($CDCl_3$, 400.13 MHz) 8.11 (s, 1H, ArH-triazole), 7.93-7.88 (m, 10H, 2xArH-triazole, 8xArH), 7.51-7.45 (m, 4H, 4xArH), 7.31-7.22 (m, 4H, 4xArH), 4.97 (d, 1H, *J* = 11.2 Hz, CH_a-3'), 4.85-4.81 (m, 4H, 1-H, CH_b-3', CH_a-2', CH_a-6'), 4.77-4.69 (m, 6H, CH_2-4', 4xNH), 4.62 (d, 1H, *J* = 12.5 Hz, CH_b-6'), 4.58 (d, 1H, *J* = 11.4 Hz, CH_b-2'), 4.46-4.42 (m, 8H, 4xCH_2), 3.85-3.80 (m, 2H, 3-H, CH_a-6), 3.69-3.64 (m, 2H, 5-H, CH_b-6), 3.54-3.46 (m, 10H, 2-H, 4-H, 4xCH_2-tacrine), 3.36 (s, 3H, OCH_3), 3.02-3.01 (m, 8H, 4xCH_2-tacrine), 2.67-2.62 (m, 8H, 4xCH_2-tacrine), 2.22-2.19 (m, 8H, 4xCH_2), 1.83 (bs, 16H, 4xCH_2-tacrine, 4xCH_2); ^{13}C NMR δ_H ($CDCl_3$, 213.76 MHz) 157.9-157.7 (4xAr), 150.8-150.7 (4xAr), 146.3-146.1 (4xAr), 145.4 (Ar-triazole), 145.0-144.9 (3xAr-triazole), 128.9 (4xAr), 127.8-127.6 (4xAr), 124.3 (5xAr), 123.9 (CH-triazole), 123.8 (CH-triazole), 123.3 (CH-triazole), 122.6-122.5 (CH-triazole, 3xAr), 120.1-120.0 (4xAr), 116.7-116.5 (Ar), 97.7 (1-C), 81.4 (3-C), 79.8 (2-C), 77.4 (4-C), 70.0 (5-C), 68.8 (6-C), 66.6 (3'-C), 65.9 (2'-C), 64.5 (6'-C), 64.9 (4'-C), 55.3 (OCH_3), 47.8-47.7 (4xCH_2), 45.4 (4xCH_2-tacrine), 33.4-33.3 (4xCH_2-tacrine), 31.6-31.5 (4xCH_2), 25.1 (4xCH_2-tacrine), 22.9 (4xCH_2-tacrine), 22.6-22.5 (4xCH_2); HRMS (ESI): Calcd. for $C_{83}H_{99}N_{20}O_6^+$ $[M + H]^+$ 1471.8051; found 1471.8047.

3.3. Compound 5a

The crude product of **5a** was purified by aluminum oxide flash column chromatography (CH_2Cl_2/MeOH/NH_4OH 99:1:0.1 → 98:2:0.1) to provide **5a** (130.0mg, 45%) as a light-yellow foam. R_f 0.26 (Aluminum oxide: CH_2Cl_2/MeOH/NH_4OH 98:2:0.1); $[\alpha]_D^{26}$ + 17 (*c* 0.35, $CHCl_3$);

^{1}H NMR δ_H ($CDCl_3$, 400.13 MHz) 7.94 (s, 1H, ArH-triazole), 7.92-7.88 (m, 8H, 8xArH), 7.80 (s, 1H, ArH-triazole), 7.77 (s, 1H, ArH-triazole), 7.59 (s, 1H, ArH-triazole), 7.54-7.50 (m, 4H, 4xArH), 7.33-7.29 (m, 4H, 4xArH), 5.01 (d, 1H, J = 11.1 Hz, CH_a-3'), 4.92 (d, 1H, J = 11.2 Hz, CH_a-2'), 4.87 (d, 1H, J = 11.3 Hz, CH_b-3'), 4.82-4.81 (m, 3H, 1-H, CH_2-4'), 4.74 (d, 1H, J = 12.5 Hz, CH_a-6'), 4.64 (d, 1H, J = 12.5 Hz, CH_b-6'), 4.61 (d, 1H, J = 11.0 Hz, CH_b-2'), 4.30-4.24 (m, 8H, 4xCH_2), 3.96 (bs, 3H, 3xNH), 3.88-3.83 (m, 1H, 3-H), 3.81-3.80 (m, 1H, CH_a-6), 3.72-3.66 (m, 2H, 5-H, CH_b-6), 3.57-3.53 (m, 2H, 2-H, 4-H), 3.42-3.38 (m, 8H, 4xCH_2-tacrine), 3.36 (s, 3H, OCH_3), 3.04 (bs, 8H, 4xCH_2-tacrine), 2.68 (bs, 8H, 4xCH_2-tacrine), 1.89-1.85 (m, 24H, 4xCH_2-tacrine, 8xCH_2), 1.62-1.55 (m, 8H, 4xCH_2), 1.39-1.31 (m, 16H, 8xCH_2); ^{13}C NMR δ_H ($CDCl_3$, 100.61 MHz) 158.6 (4xAr), 150.7 (4xAr), 147.5 (4xAr), 145.2 (Ar-triazole), 144.9 (Ar-triazole), 144.8 (Ar-triazole), 144.7 (Ar-triazole), 128.9 (4xAr), 128.4 (4xAr), 128.4 (4xAr), 123.7 (CH-triazole), 123.4 (2xCH-triazole), 123.0 (CH-triazole), 122.8 (4xAr), 120.4 (4xAr), 116.2 (4xAr), 97.8 (1-C), 81.6 (3-C), 79.8 (2-C), 77.4 (4-C), 70.1 (5-C), 68.8 (6-C), 66.7 (3'-C), 66.2 (2'-C), 65.0 (6'-C), 64.8 (4'-C), 55.3 (OCH_3), 50.2-50.1 (4xCH_2), 49.3 (4xCH_2-tacrine), 34.1 (4xCH_2-tacrine), 31.6 (4xCH_2), 30.2 (4xCH_2), 26.4-26.3 (8xCH_2), 25.0 (4xCH_2-tacrine), 23.2 (4xCH_2-tacrine), 22.9 (4xCH_2); HRMS (ESI): Calcd. for $C_{95}H_{123}N_{20}O_6^+$ $[M + H]^+$ 1639.9929; found 1639.9907.

3.4. Compound 6a

The crude product of **6a** was purified by aluminum oxide flash column chromatography (CH_2Cl_2/MeOH 99:1 → 98:2) to provide **6a** (75.1 mg, 25%) as a brown foam. R_f 0.33 (Aluminum oxide: CH_2Cl_2/MeOH/NH_4OH 98:2:0.1); $[\alpha]_D^{26} + 22$ (c 0.27, $CHCl_3$); ^{1}H NMR δ_H ($CDCl_3$, 850.13 MHz) 7.91-7.90 (m, 5H, 4xArH, ArH-triazole), 7.85 (d, 4H, J = 8.3 Hz, 4xArH), 7.79 (s, 1H, ArH-triazole), 7.75 (s, 1H, ArH-triazole), 7.58 (s, 1H, ArH-triazole), 7.49 (t, 4H, J = 7.2 Hz, 4xArH), 7.28 (t, 4H, J = 7.5 Hz, 4xArH), 5.00 (d, 1H, J = 11.1 Hz, CH_a-3'), 4.91 (d, 1H, J = 11.1 Hz, CH_a-2'), 4.86 (d, 1H, J = 11.1 Hz, CH_b-3'), 4.83-4.79 (m, 3H, 1-H, CH_2-4'), 4.72 (d, 1H, J = 12.4 Hz, CH_a-6'), 4.62 (d, 1H, J = 12.4 Hz, CH_b-6'), 4.60 (d, 1H, J = 11.1 Hz, CH_b-2'), 4.26-4.25 (m, 8H, 4xCH_2), 3.92 (bs, 4H, 4xNH), 3.84 (t, 1H, J = 9.2 Hz, 3-H), 3.80-3.79 (m, 1H, CH_a-6), 3.69 (d, 1H, J = 10.1 Hz, CH_b-6), 3.66-3.65 (m, 1H, 5-H), 3.54-3.53 (m, 2H, 2-H and 4-H), 3.40 (bs, 8H, 4xCH_2-tacrine), 3.34 (s, 3H, OCH_3), 3.01 (bs, 8H, 4xCH_2-tacrine), 2.65 (bs, 8H, 4xCH_2-tacrine), 1.86-1.83 (m, 24H, 4xCH_2-tacrine, 8xCH_2), 1.59-1.57 (m, 8H, 4xCH_2), 1.31-1.25 (m, 32H,

16xCH_2); ^{13}C NMR δ_H ($CDCl_3$, 213.76 MHz) 158.5 (4xAr), 150.7 (4xAr), 147.5 (4xAr), 145.1 (Ar-triazole), 144.7 (Ar-triazole), 144.6 (Ar-triazole), 144.6 (Ar-triazole), 128.8 (4xAr), 128.2 (4xAr), 123.6 (5xAr), 123.3 (CH-triazole), 123.2 (CH-triazole), 122.8 (4xAr), 122.7 (CH-triazole), 120.3 (4xAr), 115.9 (4xAr), 97.5 (1-C), 81.5 (3-C), 79.7 (2-C), 77.4 (4-C), 69.9 (5-C), 68.7 (6-C), 66.6 (3'-C), 66.1 (2'-C), 64.7 (6'-C), 64.5 (4'-C), 55.2 (OCH_3), 50.3-50.2 (4xCH_2), 49.4 (4xCH_2-tacrine), 34.1 (4xCH_2-tacrine), 31.7 (4xCH_2), 30.2 (4xCH_2), 29.1 (4xCH_2), 28.9-28.8 (4xCH_2), 26.8 (4xCH_2), 26.4-26.3 (4xCH_2), 24.8 (4xCH_2-tacrine), 23.1 (4xCH_2-tacrine), 22.8 (4xCH_2); HRMS (ESI): Calcd. for $C_{103}H_{139}N_{20}O_6^+$ $[M + H]^+$ 1752.1181; found 1752.1161.

4. References

[1] F. P. Balderas, J. M. Sanfrutos, F. H. Mateo, J. I. García, F. S. Gonzalez. *Eur. J. Org. Chem.* **2009**, *15*, 2441-2453.

5. NMR spectra

^{1}H-NMR spectra of compound **13** ($CDCl_3$, 400.13 MHz)

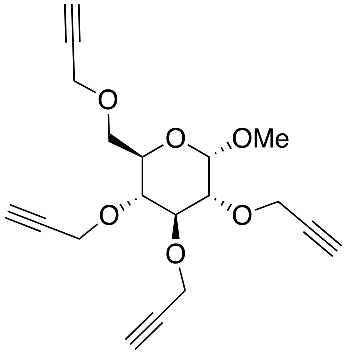

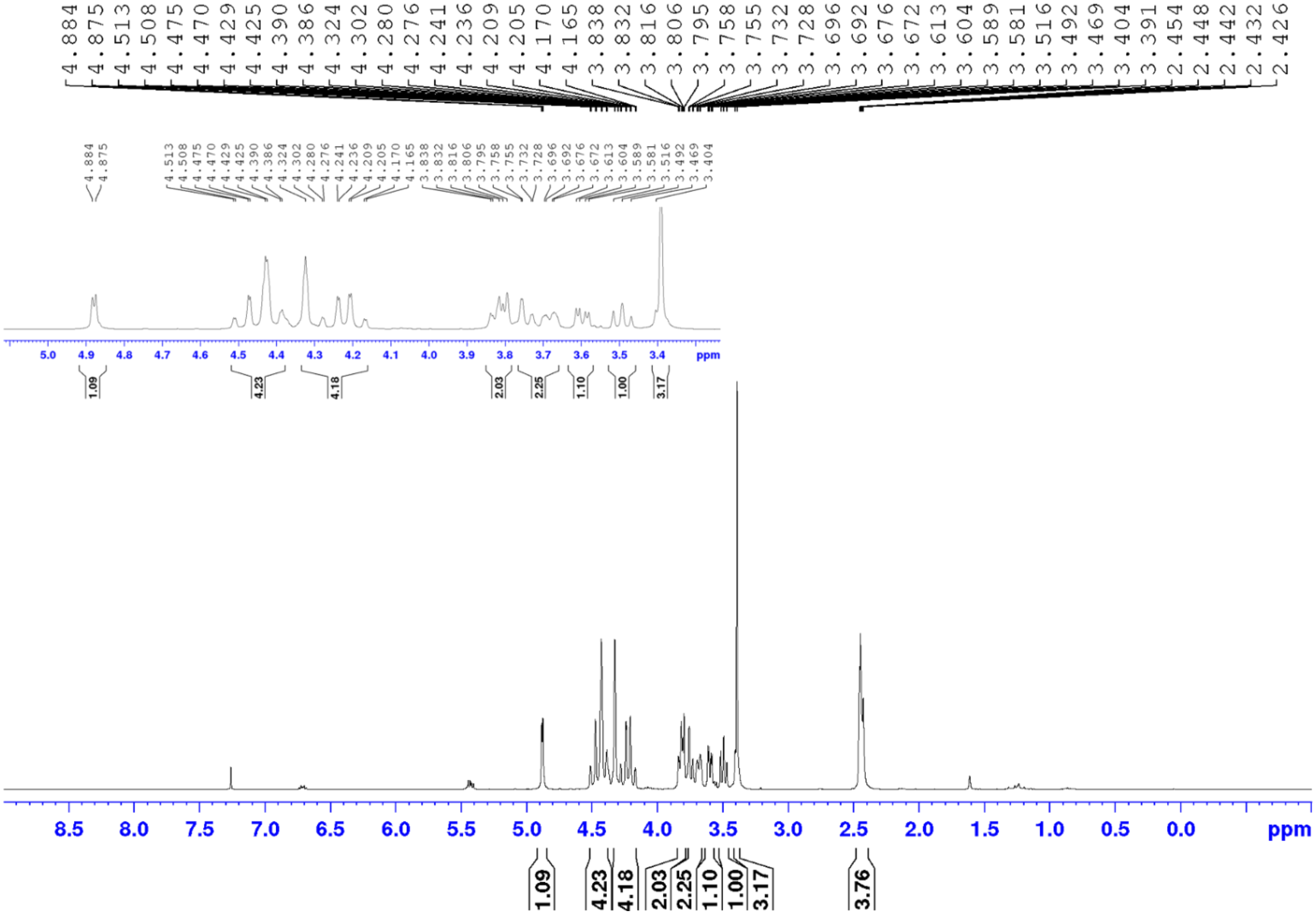

^{13}C-NMR spectra of compound **13** ($CDCl_3$, 100.61 MHz)

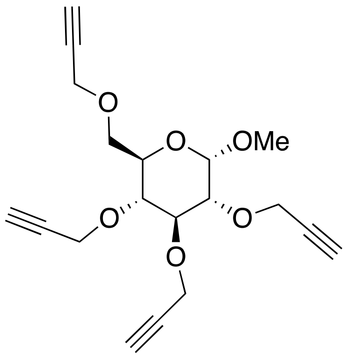

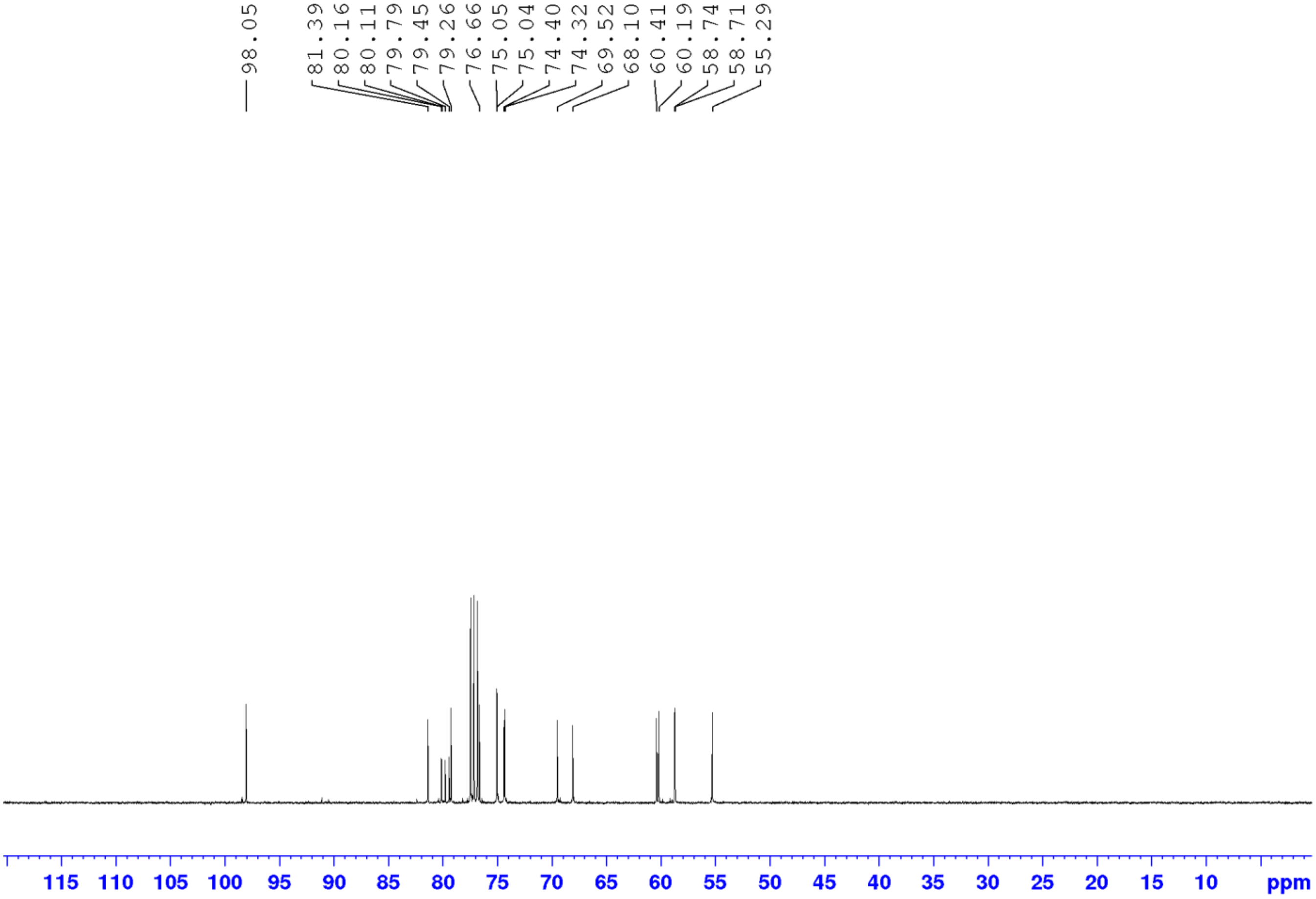

^{1}H-NMR spectra of compound **3b** (DMSO, 400.13 MHz)

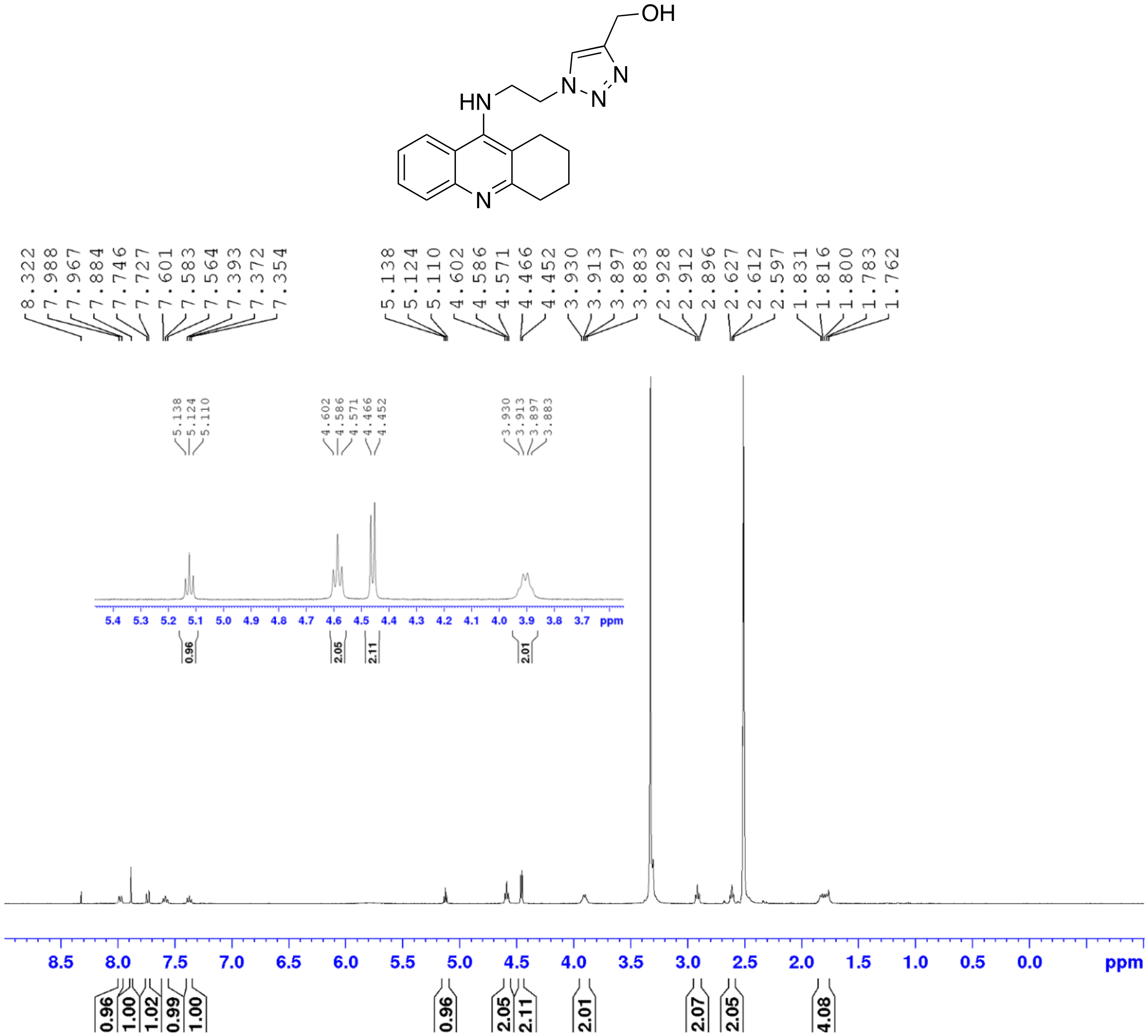

^{13}C-NMR spectra of compound **3b** (DMSO, 100.61 MHz)

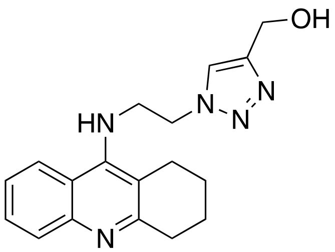

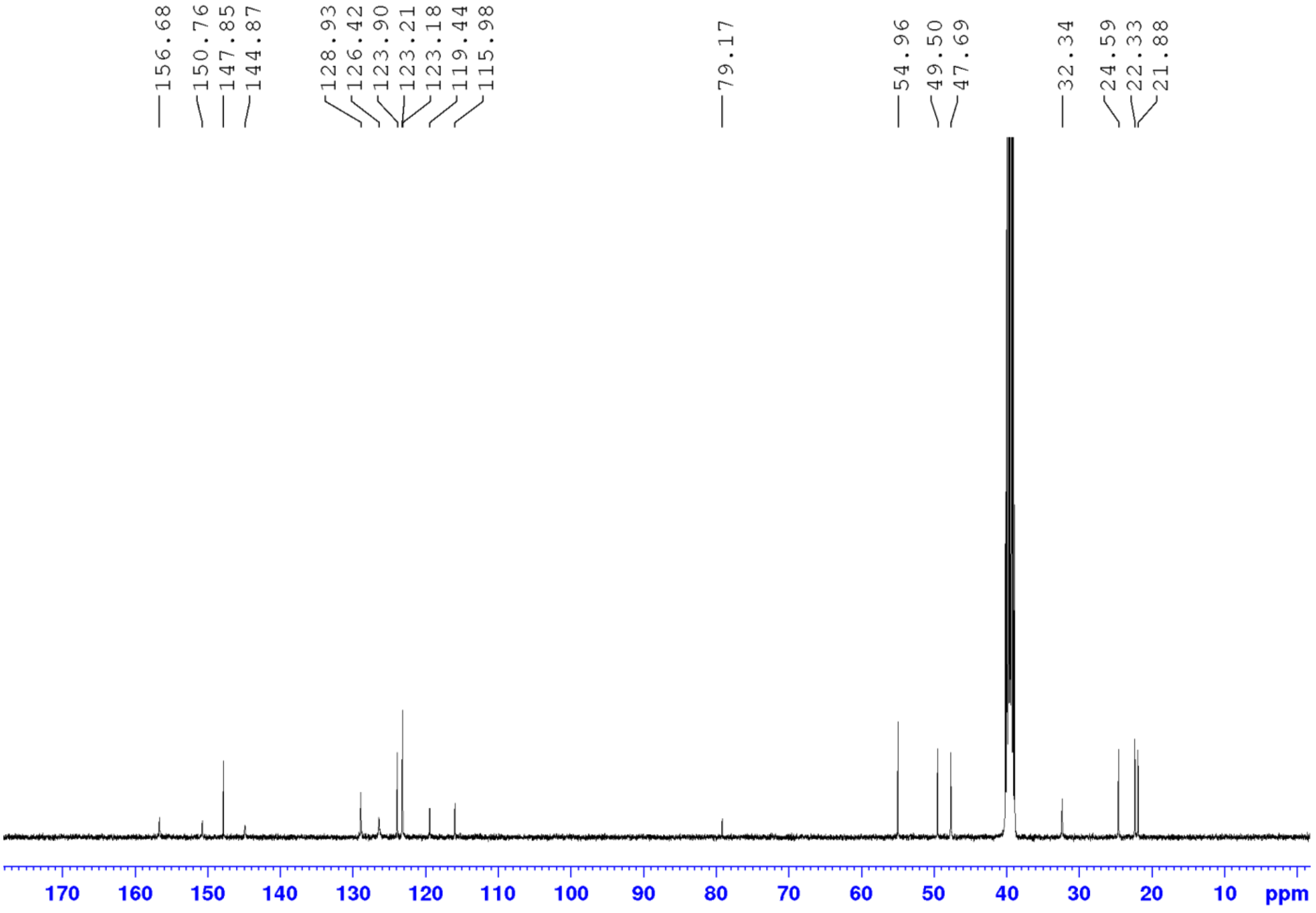

^{1}H-NMR spectra of compound 4b (MeOD, 850.13 MHz)

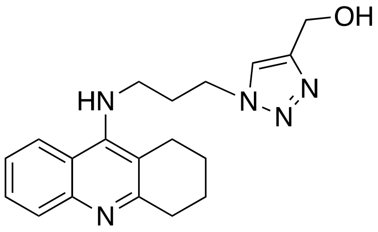

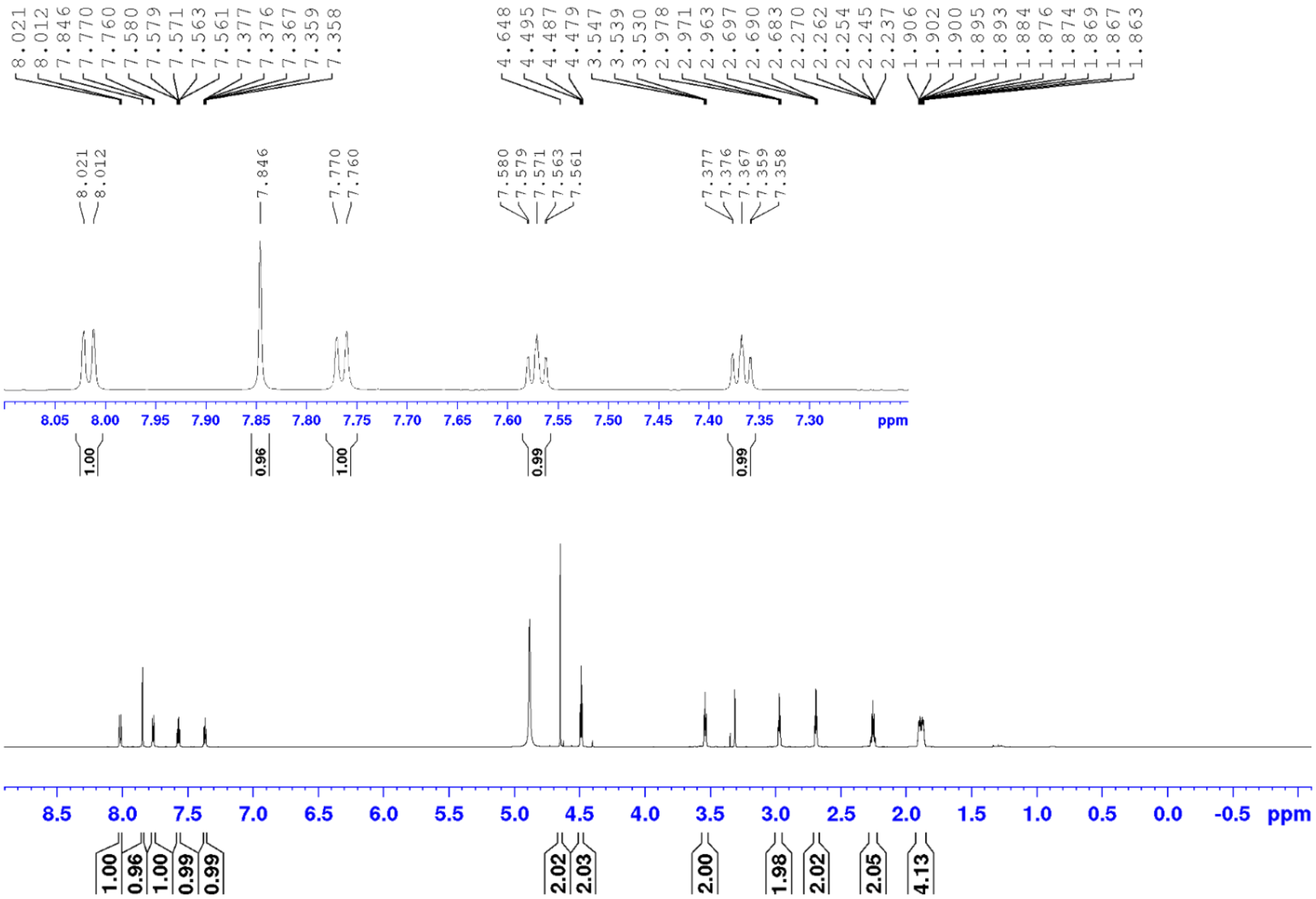

^{13}C-NMR spectra of compound **4b** (MeOD, 213.76 MHz)

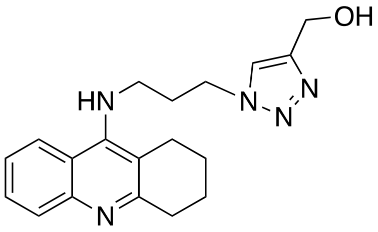

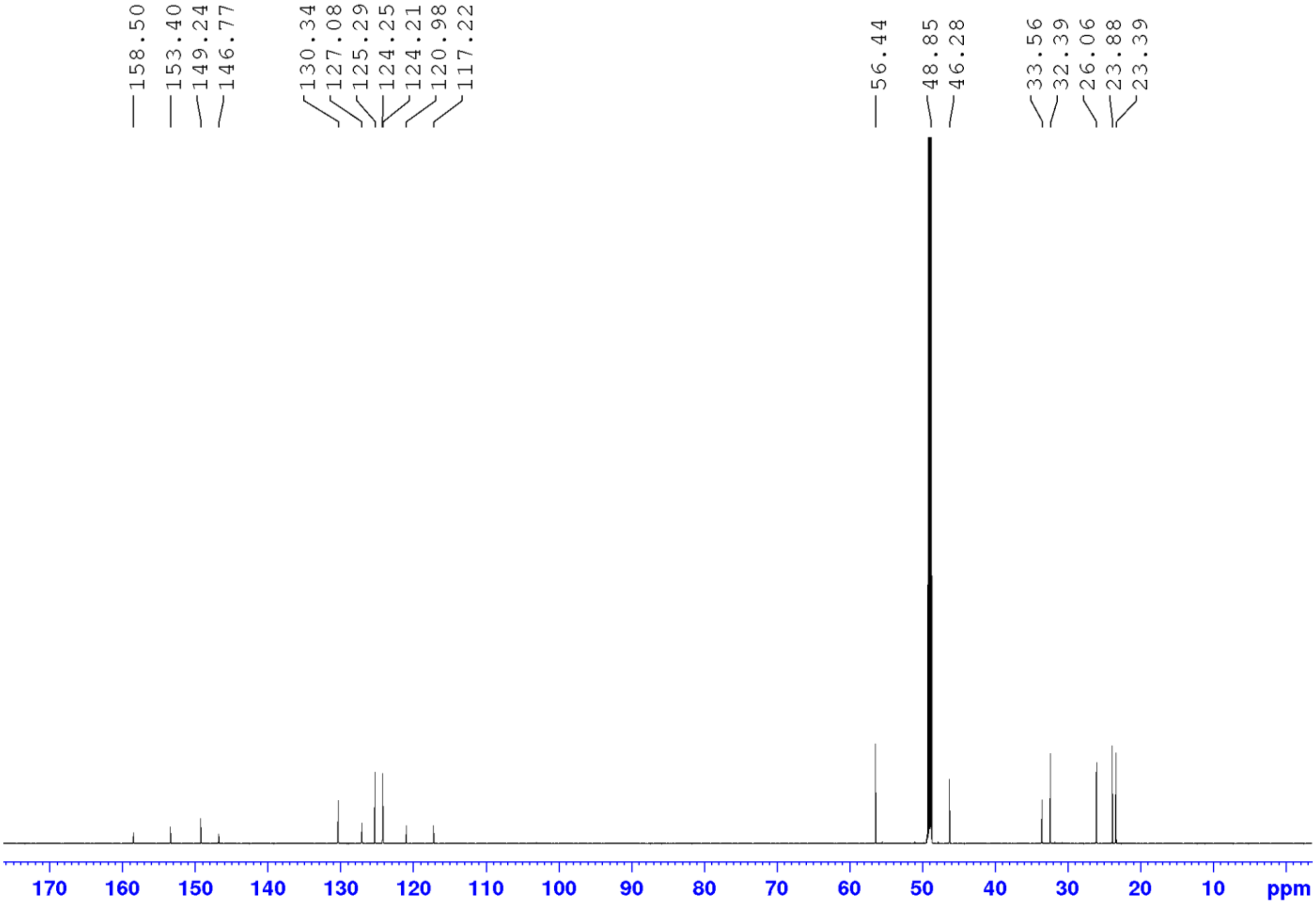

^{1}H-NMR spectra of compound **5b** (MeOD, 400.13 MHz)

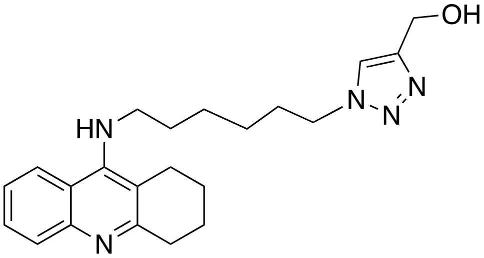

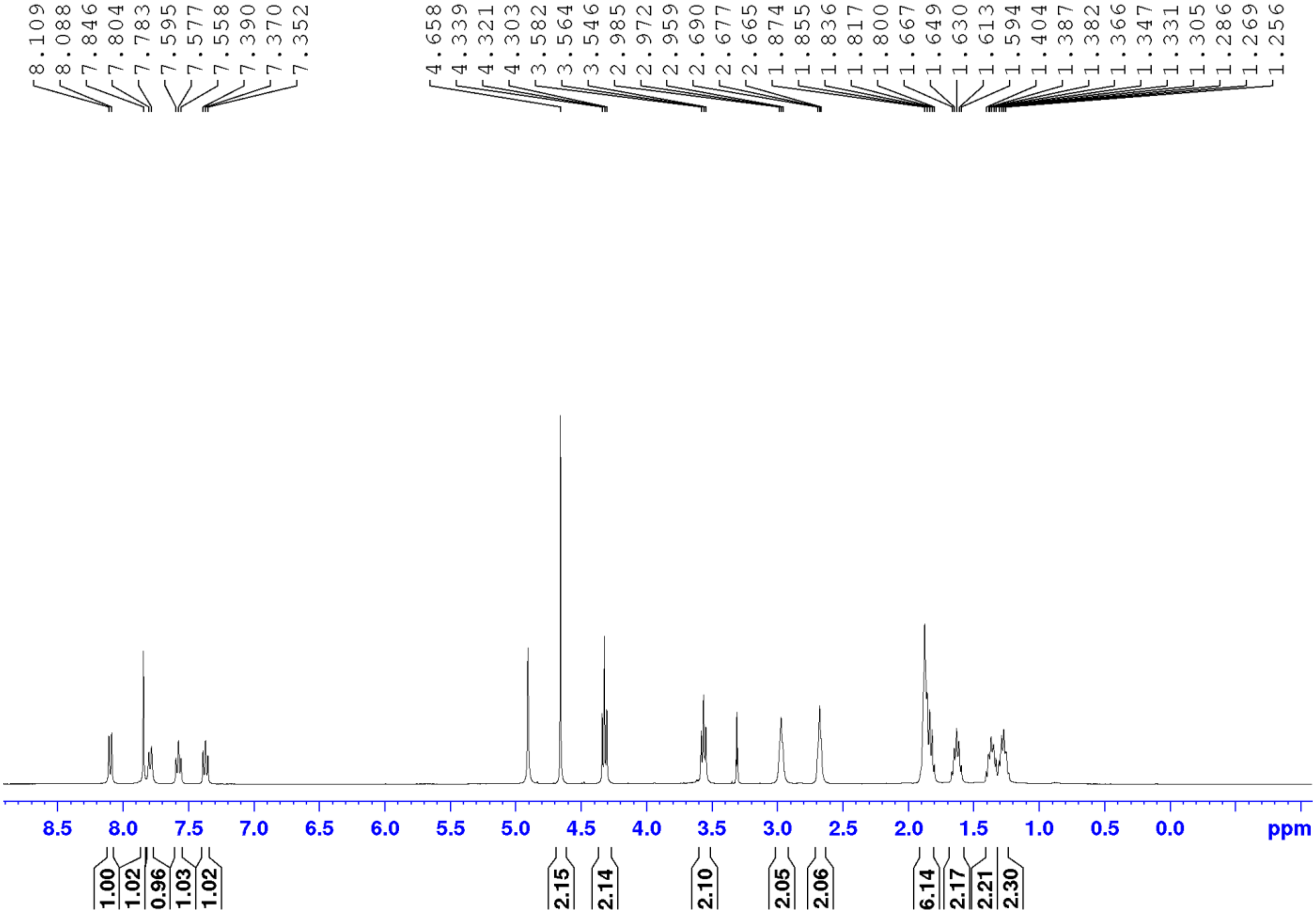

^{13}C-NMR spectra of compound **5b** (MeOD, 100.61 MHz)

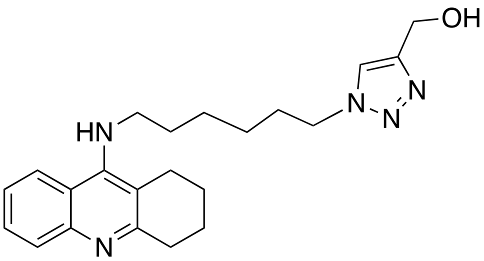

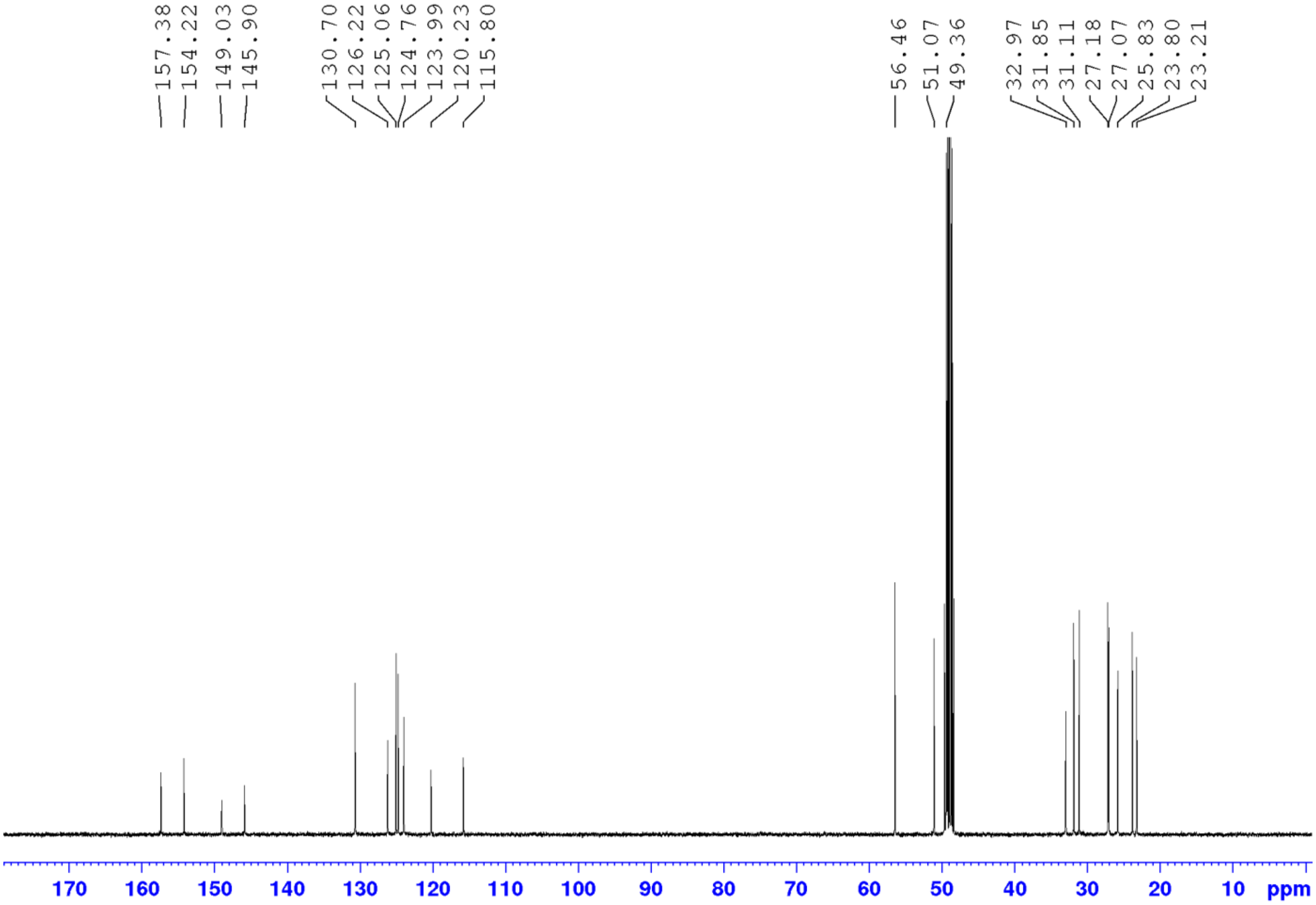

^{1}H-NMR spectra of compound **6b** (MeOD, 400.13 MHz)

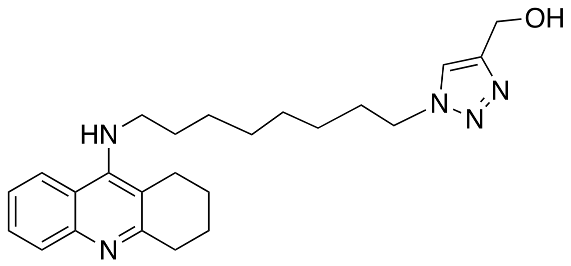

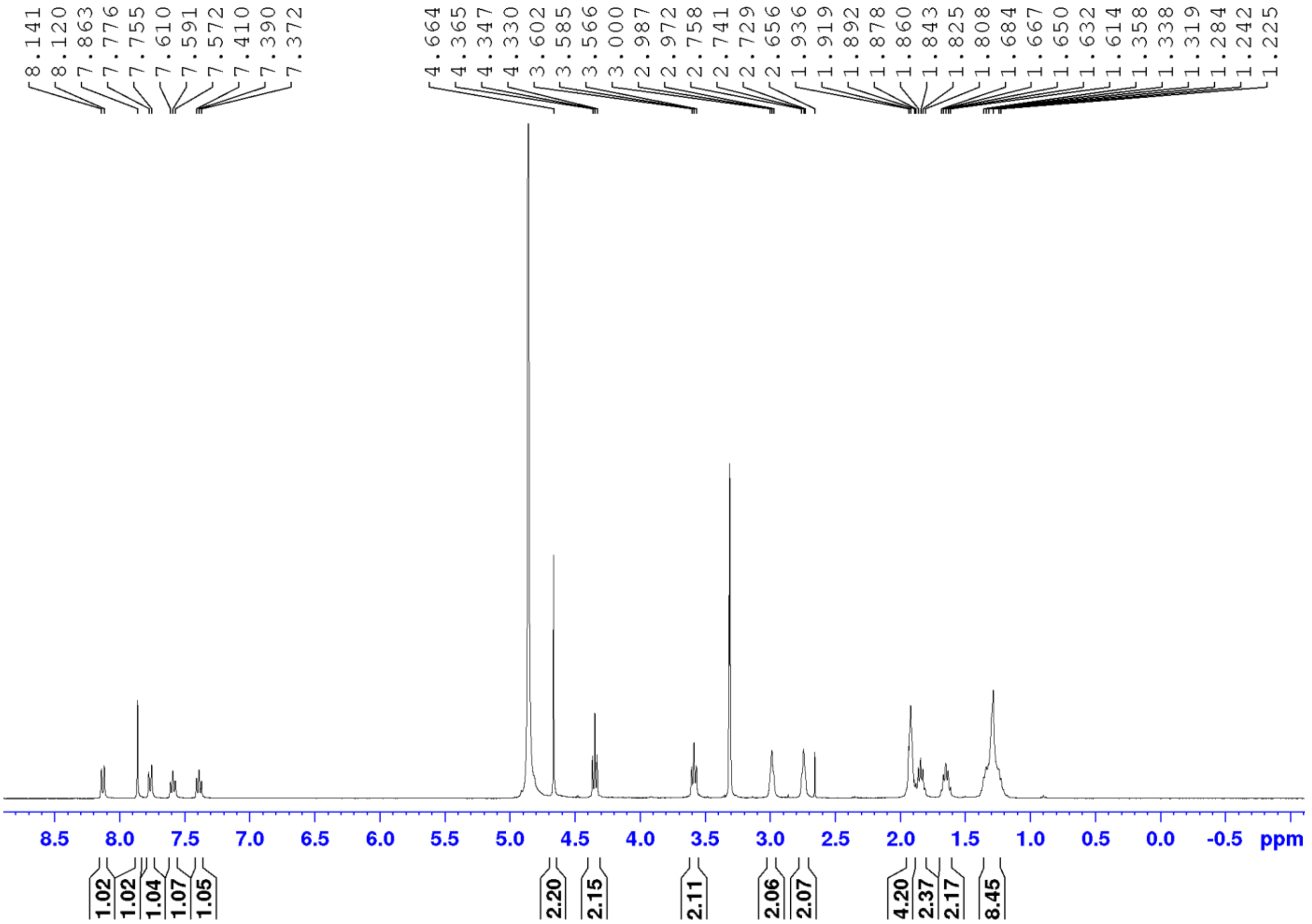

^{13}C-NMR spectra of compound **6b** (MeOD, 100.61 MHz)

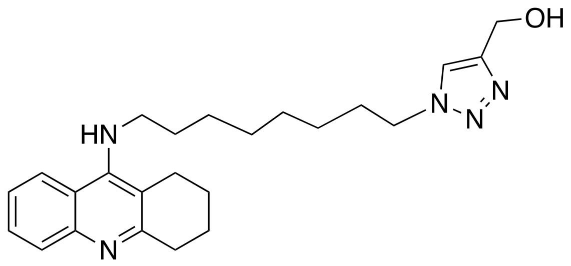

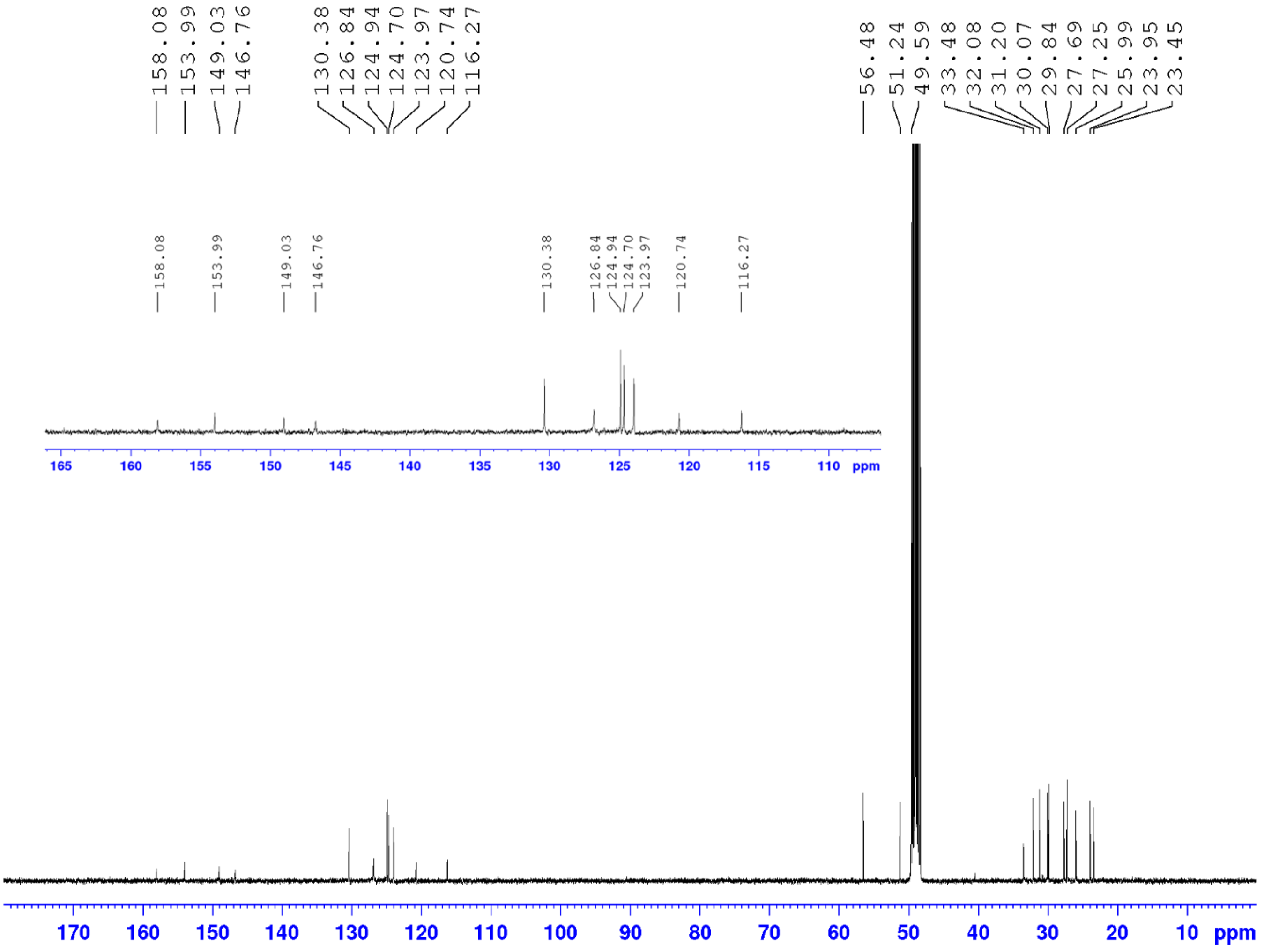

^{1}H-NMR spectra of compound **3a** ($CDCl_3$, 850.13 MHz)

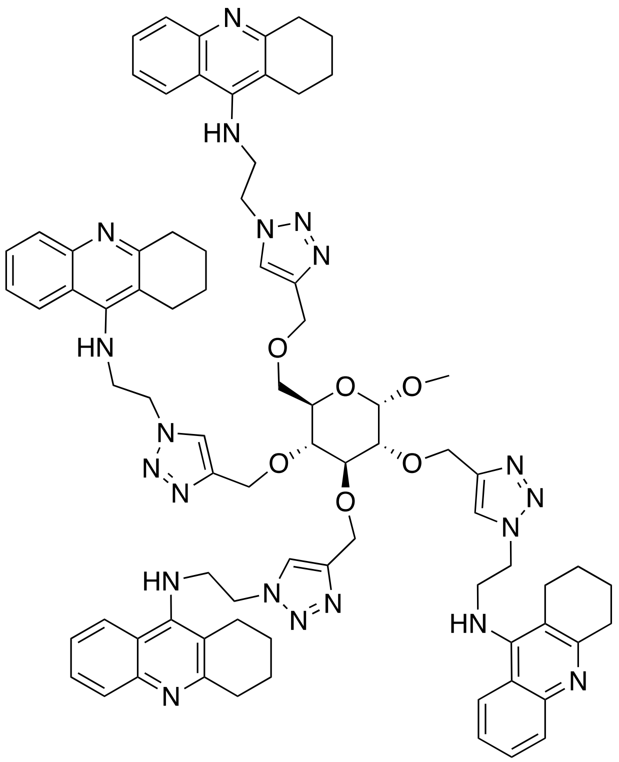

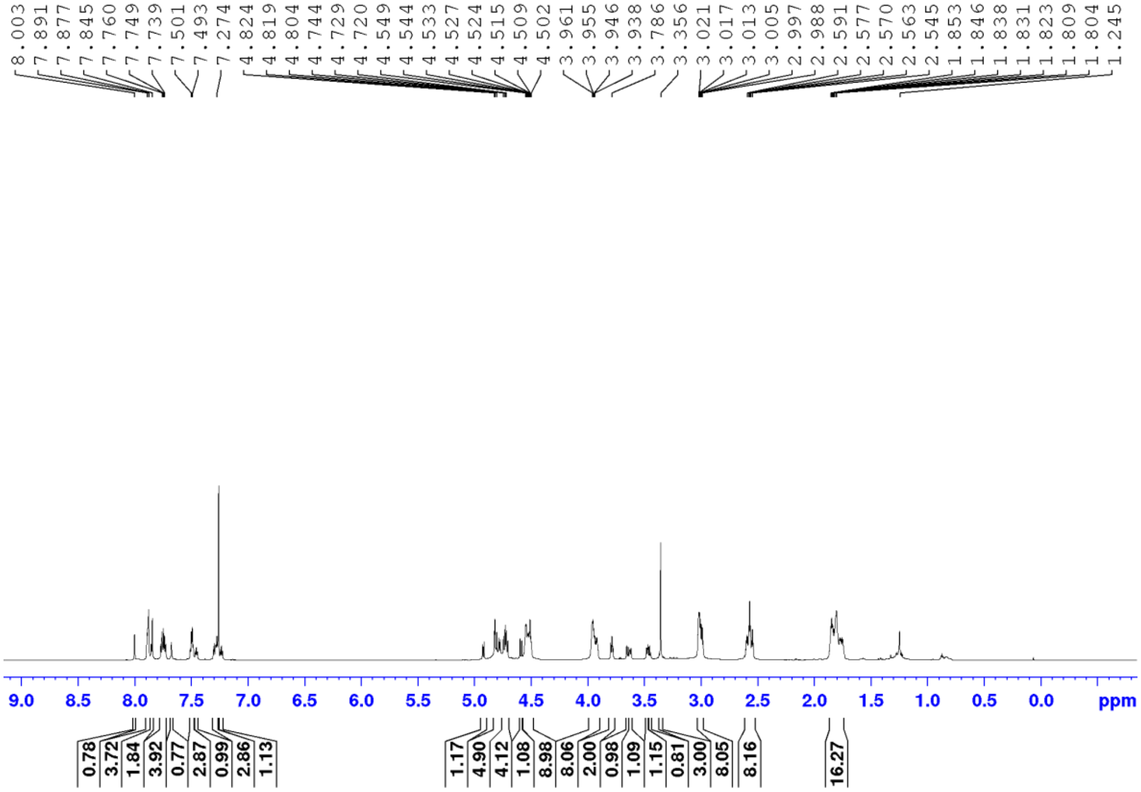

^{13}C-NMR spectra of compound **3a** ($CDCl_3$, 213.76 MHz)

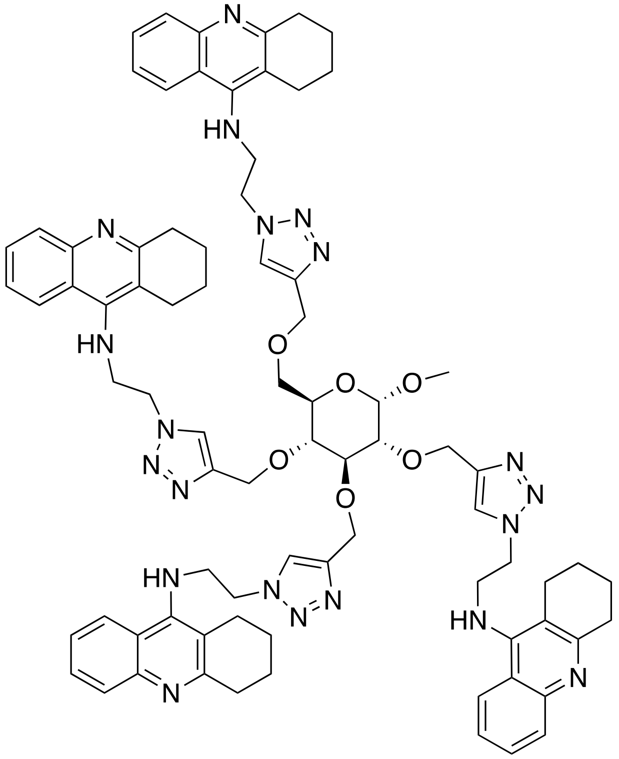

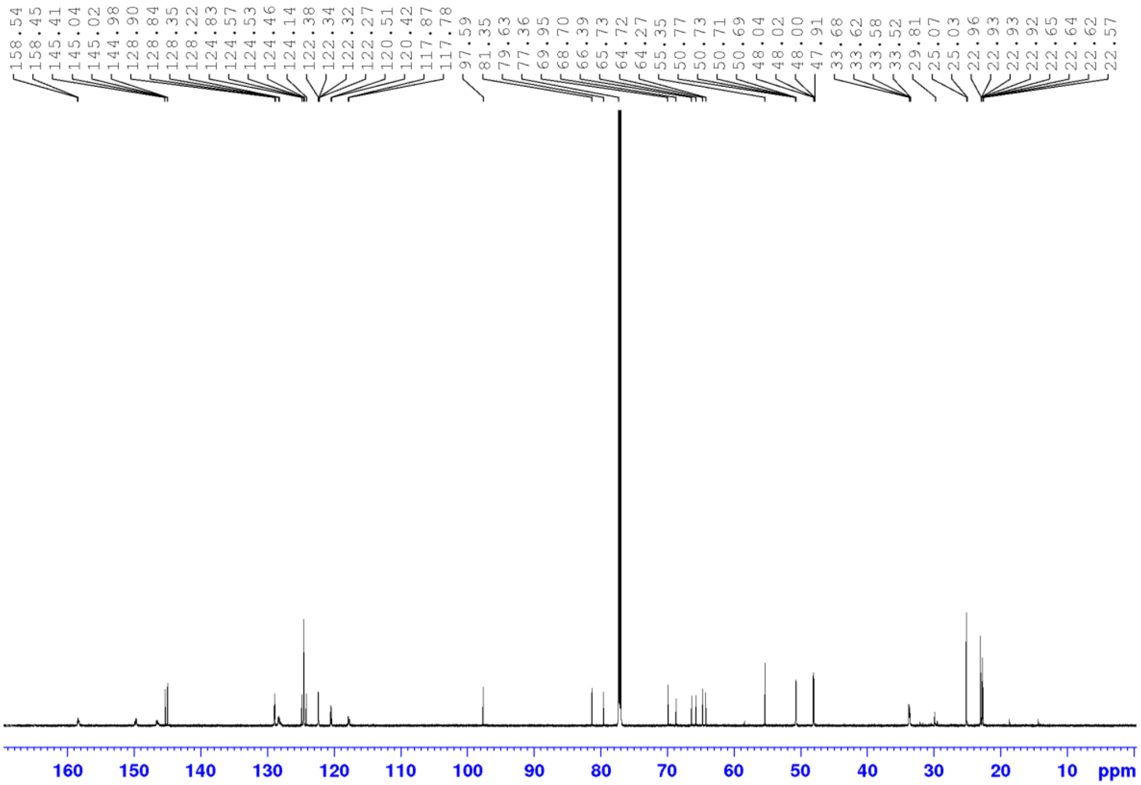

^{1}H-NMR spectra of compound **4a** ($CDCl_3$, 400.13 MHz)

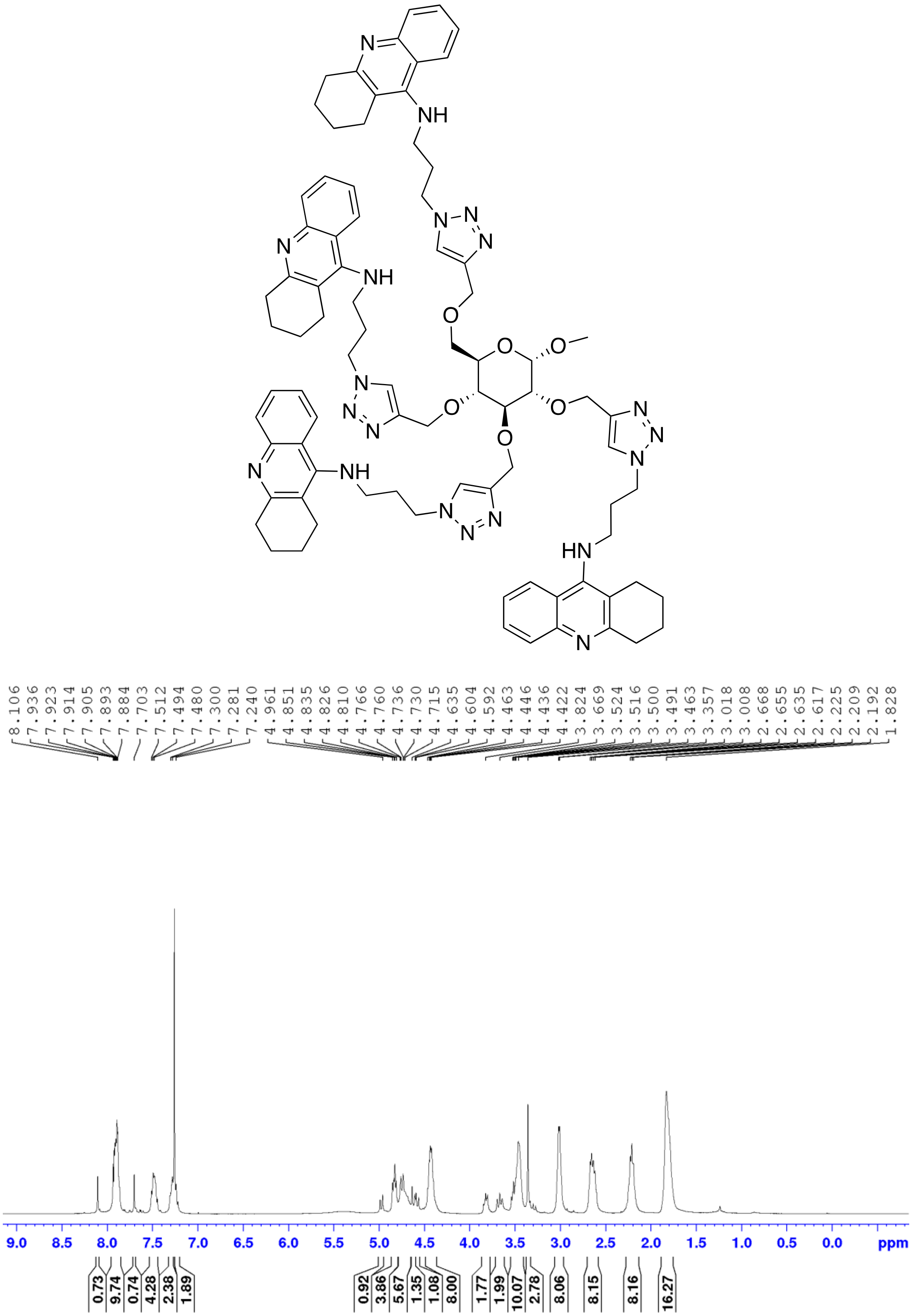

^{13}C-NMR spectra of compound **4a** ($CDCl_3$, 100.61 MHz)

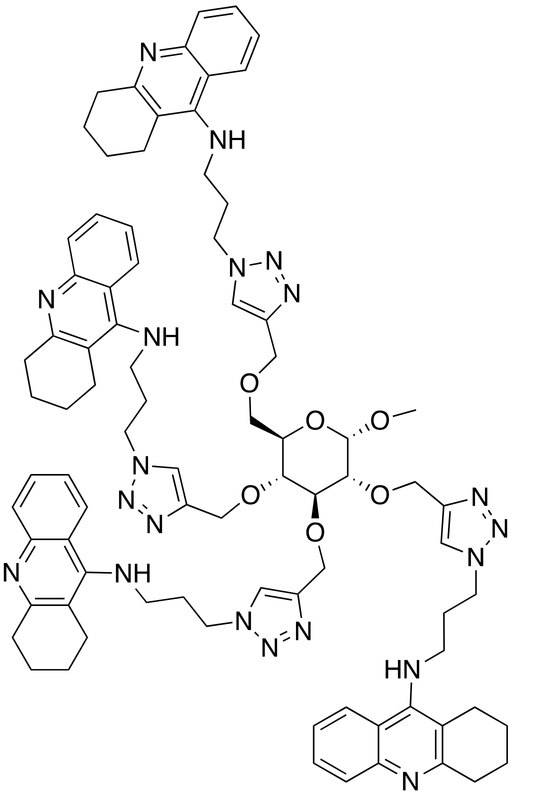

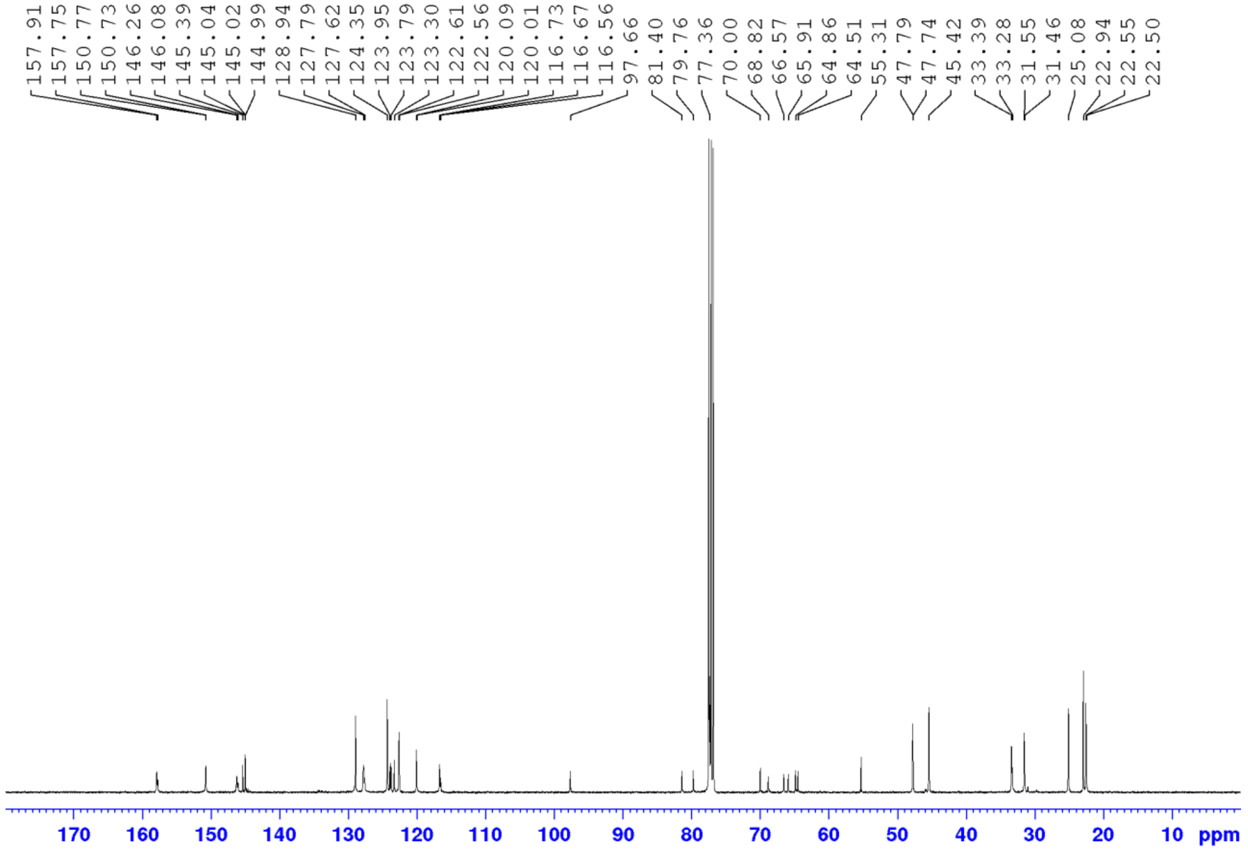

^{1}H-NMR spectra of compound **5a** ($CDCl_3$, 400.13 MHz)

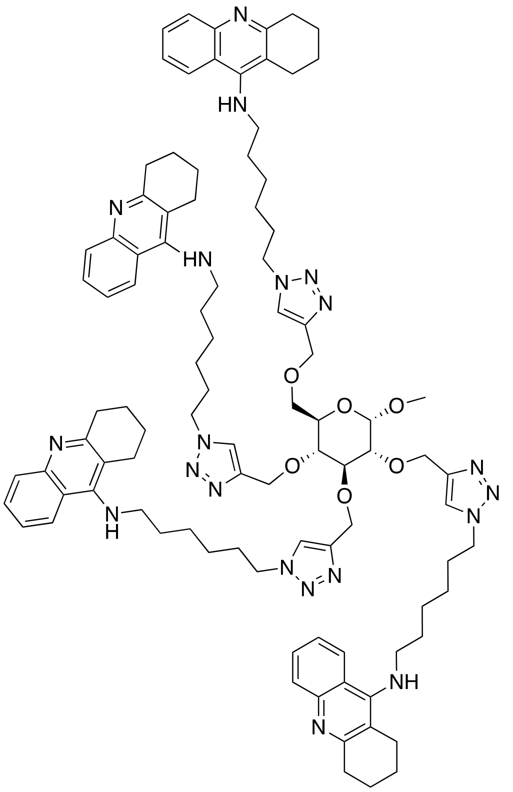

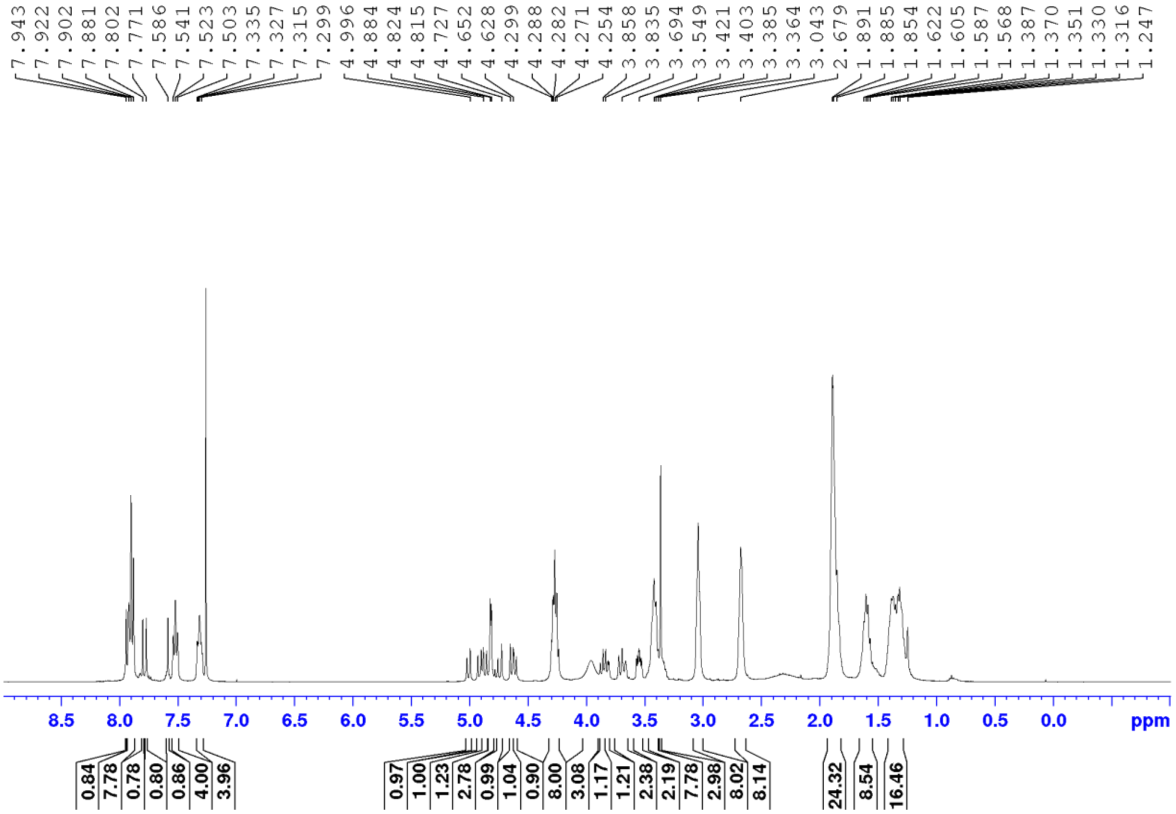

^{13}C-NMR spectra of compound **5a** ($CDCl_3$, 100.61 MHz)

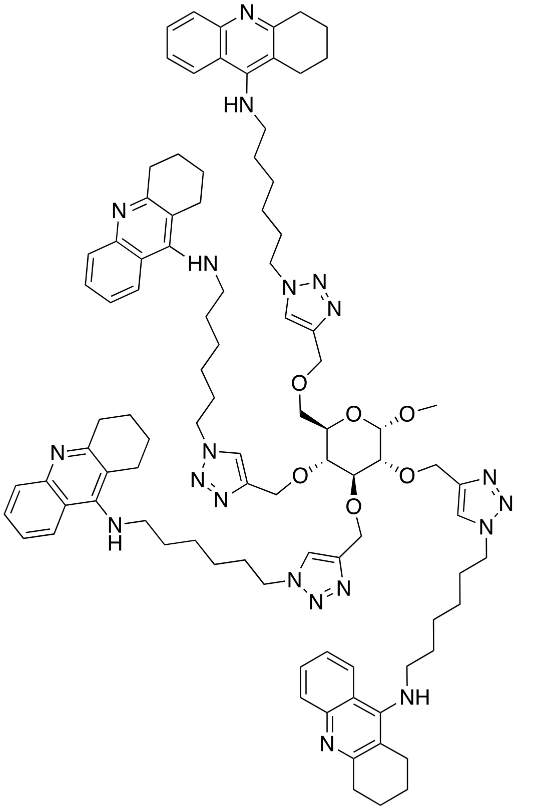

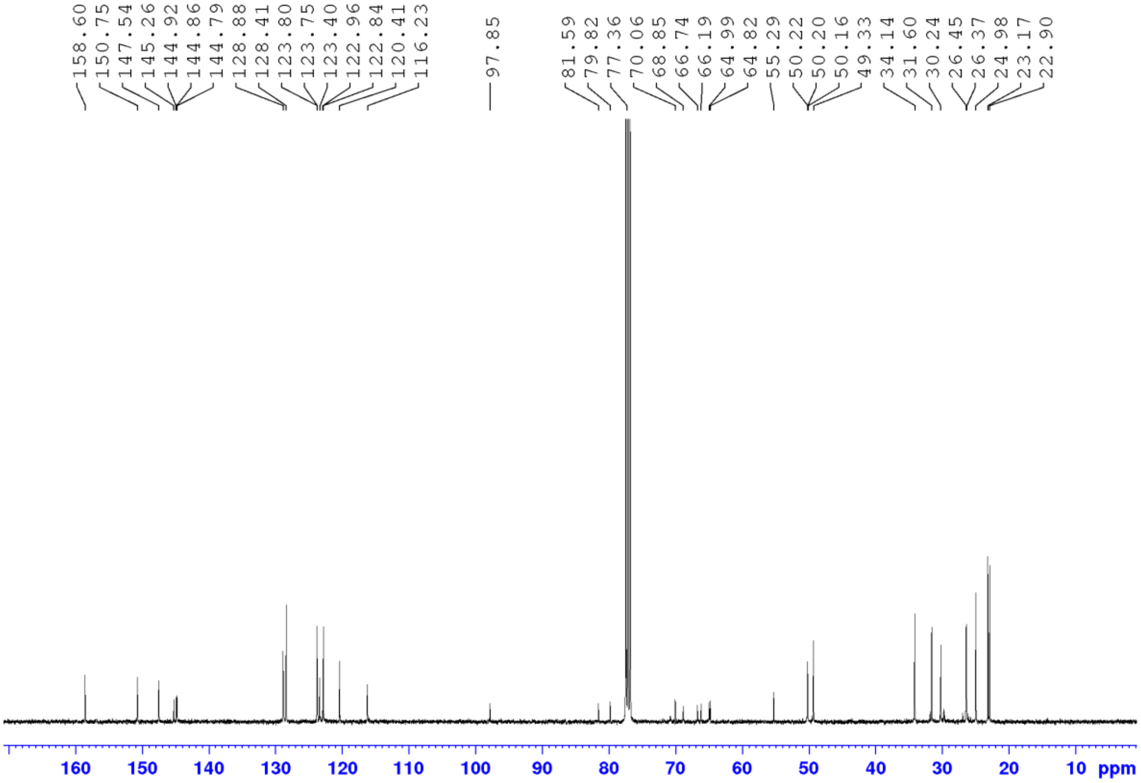

^{1}H-NMR spectra of compound **6a** ($CDCl_3$, 850.13 MHz)

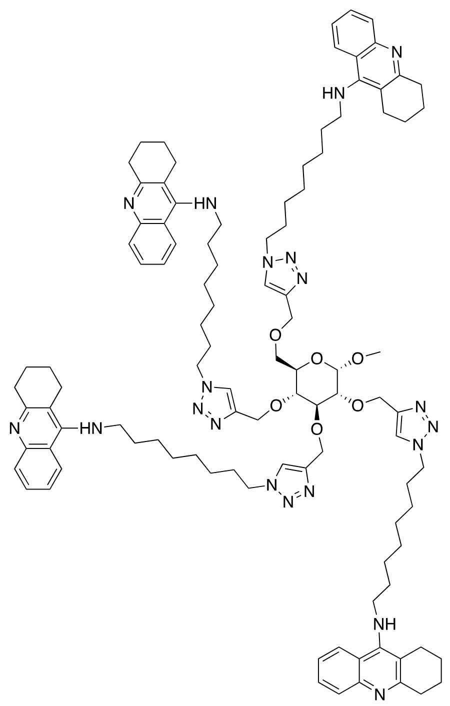

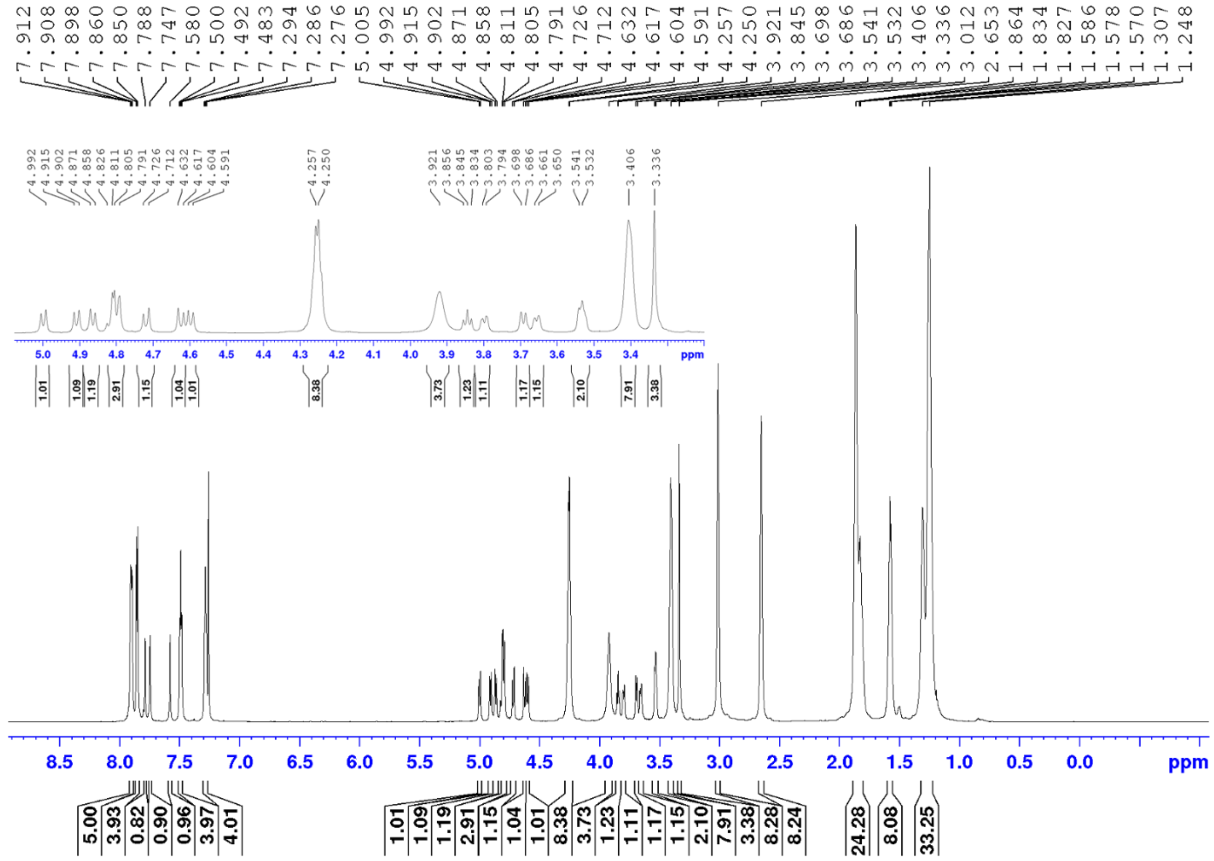

^{13}C-NMR spectra of compound **6a** ($CDCl_3$, 213.76 MHz)

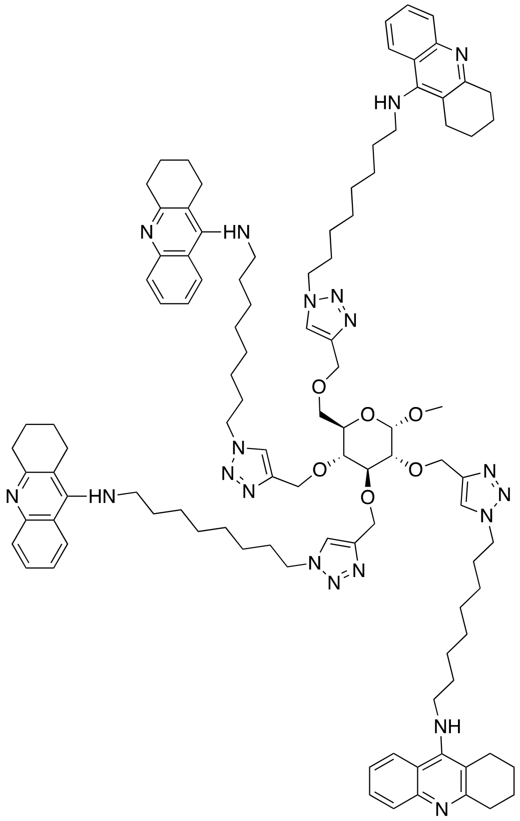

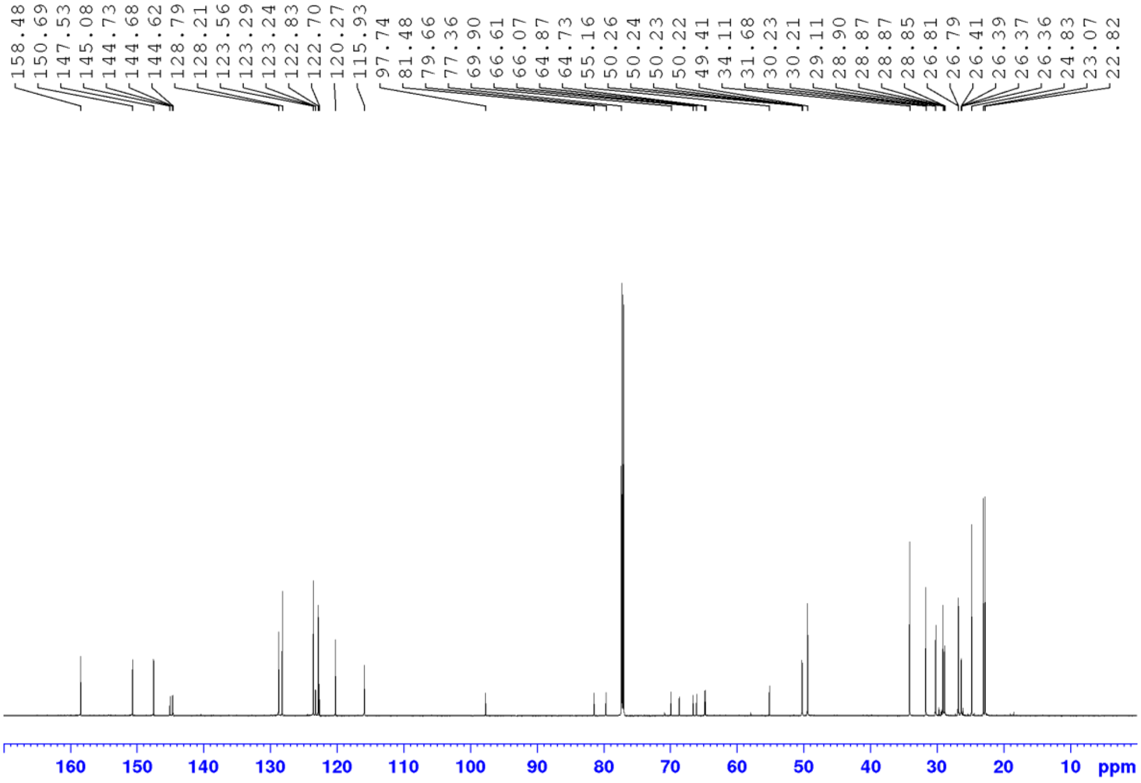

Supplement: Supplemental Material [file IENZ_A_1954917_SM5802.pdf]
